# Family‐centred care interventions for children with chronic conditions: A scoping review

**DOI:** 10.1111/hex.13897

**Published:** 2024-02-02

**Authors:** Andrea J. Chow, Ammar Saad, Zobaida Al‐Baldawi, Ryan Iverson, Becky Skidmore, Isabel Jordan, Nicole Pallone, Maureen Smith, Pranesh Chakraborty, Jamie Brehaut, Eyal Cohen, Sarah Dyack, Jane Gillis, Sharan Goobie, Cheryl R. Greenberg, Robin Hayeems, Brian Hutton, Michal Inbar‐Feigenberg, Shailly Jain‐Ghai, Sara Khangura, Jennifer J. MacKenzie, John J. Mitchell, Zeinab Moazin, Stuart G. Nicholls, Amy Pender, Chitra Prasad, Andreas Schulze, Komudi Siriwardena, Rebecca N. Sparkes, Kathy N. Speechley, Sylvia Stockler, Monica Taljaard, Mari Teitelbaum, Yannis Trakadis, Clara Van Karnebeek, Jagdeep S. Walia, Kumanan Wilson, Beth K. Potter

**Affiliations:** ^1^ School of Epidemiology and Public Health University of Ottawa Ottawa Ontario Canada; ^2^ Ottawa Ontario Canada; ^3^ Squamish British Columbia Canada; ^4^ Sparwood British Columbia Canada; ^5^ Canadian Organization for Rare Disorders Ottawa Ontario Canada; ^6^ Newborn Screening Ontario, Children's Hospital of Eastern Ontario Ottawa Ontario Canada; ^7^ Department of Pediatrics University of Ottawa Ottawa Ontario Canada; ^8^ Clinical Epidemiology Program Ottawa Hospital Research Institute Ottawa Ontario Canada; ^9^ Department of Pediatrics University of Toronto/Hospital for Sick Children Toronto Ontario Canada; ^10^ Department of Pediatrics Dalhousie University Halifax Nova Scotia Canada; ^11^ Moffit Cancer Care Center Tampa Florida USA; ^12^ Department of Pediatrics and Child Health University of Manitoba Winnipeg Manitoba Canada; ^13^ Child Health Evaluative Sciences University of Toronto/Hospital for Sick Children Toronto Ontario Canada; ^14^ Division of Clinical & Metabolic Genetics Hospital for Sick Children Toronto Ontario Canada; ^15^ Department of Pediatrics University of Toronto Toronto Ontario Canada; ^16^ Department of Medical Genetics University of Alberta Edmonton Alberta Canada; ^17^ Department of Pediatrics McMaster University Hamilton Ontario Canada; ^18^ Department of Medicine Queen's University Kingston Ontario Canada; ^19^ Montreal Children's Hospital Montreal Quebec Canada; ^20^ McMaster Children's Hospital Hamilton Ontario Canada; ^21^ Department of Pediatrics Western University London Ontario Canada; ^22^ Department of Biochemistry and Department of Pediatrics University of Toronto Toronto Ontario Canada; ^23^ Clinical and Metabolic Genetics Hospital for Sick Children Toronto Ontario Canada; ^24^ Alberta Children's Hospital Calgary Alberta Canada; ^25^ Departments of Pediatrics and Epidemiology and Biostatistics Western University London Ontario Canada; ^26^ BC Children's Hospital Vancouver British Columbia Canada; ^27^ Children's Hospital of Eastern Ontario Ottawa Ontario Canada; ^28^ Departments of Pediatrics and Human Genetics Emma Center for Personalized Medicine, Amsterdam UMC Amsterdam The Netherlands; ^29^ Department of Pediatrics Queen's University Kingston Ontario Canada; ^30^ Department of Medicine University of Ottawa Ottawa Ontario Canada; ^31^ Bruyère Research Institute Ottawa Ontario Canada

**Keywords:** chronic conditions, family‐centred care, paediatrics, quality improvement, scoping review

## Abstract

**Introduction:**

Children with chronic conditions have greater health care needs than the general paediatric population but may not receive care that centres their needs and preferences as identified by their families. Clinicians and researchers are interested in developing interventions to improve family‐centred care need information about the characteristics of existing interventions, their development and the domains of family‐centred care that they address. We conducted a scoping review that aimed to identify and characterize recent family‐centred interventions designed to improve experiences with care for children with chronic conditions.

**Methods:**

We searched Medline, Embase, PsycInfo and Cochrane databases, and grey literature sources for relevant articles or documents published between 1 January 2019 and 11 August 2020 (databases) or 7–20 October 2020 (grey literature). Primary studies with ≥10 participants, clinical practice guidelines and theoretical articles describing family‐centred interventions that aimed to improve experiences with care for children with chronic conditions were eligible. Following citation and full‐text screening by two reviewers working independently, we charted data covering study characteristics and interventions from eligible reports and synthesized interventions by domains of family‐centred care.

**Results:**

Our search identified 2882 citations, from which 63 articles describing 61 unique interventions met the eligibility criteria and were included in this review. The most common study designs were quasiexperimental studies (*n* = 18), randomized controlled trials (*n* = 11) and qualitative and mixed‐methods studies (*n* = 9 each). The most frequently addressed domains of family‐centred care were communication and information provision (*n* = 45), family involvement in care (*n* = 37) and access to care (*n* = 30).

**Conclusion:**

This review, which identified 61 unique interventions aimed at improving family‐centred care for children with chronic conditions across a range of settings, is a concrete resource for researchers, health care providers and administrators interested in improving care for this high‐needs population.

**Patient or Public Contribution:**

This study was co‐developed with three patient partner co‐investigators, all of whom are individuals with lived experiences of rare chronic diseases as parents and/or patients and have prior experience in patient engagement in research (I. J., N. P., M. S.). These patient partner co‐investigators contributed to this study at all stages, from conceptualization to dissemination.

## 1 INTRODUCTION

Children with chronic health conditions or disabilities have elevated health care needs, interact with health care systems more frequently or intensively over time and often have more unmet needs relative to the general paediatric population.^1^, ^2^, ^3^, ^4^ Health care systems are often ill‐equipped to respond effectively to the needs of this population, which may include interdisciplinary services, preventive care and timely interventions to support development.^5^, ^6^ It is now an accepted standard that health care be family‐centred, particularly for children with chronic conditions.^7^, ^8^ Family‐centred care refers to practices that engage patients and their families as integral, valued members involved in decision‐making and appraisal of their health care, and may contribute to higher levels of family satisfaction with care and lower levels of distress.^9^, ^10^, ^11^ In paediatrics, family‐centred care recognizes that the family is the primary unit of support for a child's health care management and overall development.^12^ Although there is no single definition of the term nor consensus on its processes or practices, the guiding principles of family‐centred care encompasses health care policies, strategies and interventions that treat patients and their families with dignity, promote collaboration with patients and families and focus on respectful interactions.^13^, ^14^, ^15^, ^16^, ^17^ Meaningful engagement of patients and families in the co‐design or study of interventions that affect them is consistent with a family‐centred care approach and may improve intervention effectiveness and family satisfaction.^18^, ^19^

Confronted with a broadly defined topic, those interested in developing interventions to make care more family‐centred, such as policymakers, clinicians, and researchers and representatives of health care systems, may seek to learn more about, and build upon, existing interventions relevant to the health care setting of their interest. To adapt or build from such existing interventions, it would be important to understand how these similar interventions were developed, the key elements of the interventions and the aspects of family‐centred care that the interventions aim to improve. A scoping review by King and colleagues sought to identify ‘family‐oriented’ services that addressed the needs of parents of children with disabilities in paediatric rehabilitation in articles published between 2009 and 2014, leading to the development of a conceptual framework.^20^ The authors described four main types of family‐oriented interventions for parents: education about disabilities and training to administer therapy; support groups; psychosocial interventions (e.g., counselling, coaching); and provision of information about disability and available resources.^20^ Their proposed framework for family‐oriented services included six domains: information resources; education services; training/instruction services;, support groups; psychosocial services; and service coordination.

Building on King and colleagues' review, our focus for this scoping review was somewhat broader. Our overall aim was to identify and characterize recently published family‐centred health care interventions across a range of health care settings (including but not limited to rehabilitation settings) that sought to improve experiences with health care for children with chronic conditions and their families. More specifically, we sought to describe: (i) how the interventions were developed; (ii) which groups (children, caregivers, health care providers) were engaged in their development; (iii) the domains of family‐centred care that the interventions were designed to address; and (iv) the key components of the interventions that sought to address those domains.

With respect to domains of family‐centred care, we used an existing framework, the Picker principles,^21^, ^22^ as a guide. This set of principles was developed by the US Picker Institute to advocate for the needs and preferences of patients and families in health care interactions.^23^ It has eight domains (access to care, coordination, communication and information provision, family involvement, respect for child and family, follow‐up and continuity of care, physical comfort and emotional support) and has been used or adapted in studies of family experiences with health care.^24^, ^25^, ^26^

## 2 METHODS

Scoping reviews are ideal for characterizing a broad landscape of literature on a particular topic, aligned with our goal.^27^, ^28^ Our scoping review methods followed the Joanna Briggs Institute (JBI) Methodology for Scoping Reviews^29^ and our published protocol (available at https://osf.io/cjyd9/?view_only=0077822cf9ef424290651ed7b5c80177). We report our review in accordance with the Preferred Reporting Items for Systematic Reviews and Meta‐Analyses (PRISMA) extension for scoping reviews (PRISMA‐ScR) (Appendix S1).^30^

### 2.1 Patient and family engagement

Family caregivers of children with chronic conditions interact frequently with the health care system, becoming experts in their children's care needs.^31^, ^32^ Their partnership and engagement in research, especially about family‐centred care, can be expected to facilitate research questions that are more meaningful and relevant to children and their families and contribute to successful care improvement efforts.^33^, ^34^ This study was co‐developed with three patient partner co‐investigators, all of whom are individuals with lived experiences of rare chronic diseases as parents or patients and have prior experience in patient engagement in research (I. J., N. P., M. S.). These patient partner co‐investigators contributed to this study at all stages, from conceptualization to dissemination. Specifically, they participated in decision‐making about the concept of the review and its scope, contributed to the interpretation of the extracted data and critically reviewed the final manuscript.

### 2.2 Search strategy and information sources

An experienced medical information specialist (B. S.) developed and tested the search strategies through an iterative process in consultation with the research team, including review of a pre‐existing, extensive strategy covering a range of similar chronic conditions.^35^ Another senior information specialist peer reviewed the strategies before execution using the PRESS Checklist.^36^ Suggestions were reviewed and if applicable, incorporated into the final strategy. Our strategy was structured and designed to emphasize sensitivity over specificity (Appendix S2). Using the multifile option and deduplication tool on the OVID platform, we searched Ovid MEDLINE® ALL, including Epub Ahead of Print, In‐Process & Other Non‐Indexed Citations, Embase Classic+Embase, APA PsycInfo, and the following EBM Reviews Databases: Cochrane Central Register of Controlled Trials, Cochrane Database of Systematic Reviews, Database of Abstracts of Reviews of Effects, Health Technology Assessment and the NHS Economic Evaluation Database (Appendix S2). We performed all searches on 11 August 2020. Results were downloaded and deduplicated using EndNote version X9.3.3 (Clarivate Analytics) and uploaded to Covidence for screening. The number of records generated by the database search (>15,000) exceeded the study team's resources to adequately screen those records. Before screening, we made a decision to restrict articles to those published 2019 or later rather than modifying the search by further restricting the population, concept or context criteria. This restriction returned a number of records that we deemed feasible to review.

Strategies used a combination of controlled vocabulary (e.g., ‘Disabled Children’, ‘Chronic Disease’, ‘Child Health Services’) and keywords (e.g., ‘complex need’, ‘partnership’, ‘paediatric program’). Vocabulary and syntax were adjusted across the databases. Where possible, animal‐only, opinion pieces and conference abstracts were excluded from the searches. To promote the retrieval of all relevant articles, we did not limit outcomes or study designs.

We also conducted a grey literature search. Two authors (B. K. P., A. J. C.) identified pertinent resources, including the websites ClinicalTrials.gov, the International Clinical Trials Registry Platform search portal, the Patient‐Centered Outcomes Research Institute and websites recommended by CADTH's Grey matters tool.^37^ One reviewer (A. J. C.) conducted the grey literature search, performed 7–20 October 2020. A set of keywords and filters were adapted from the electronic database search strategy and tailored to the search capabilities and content of each grey literature source. Keywords included ‘child’, ‘family’, ‘caregiver’, ‘intervention’, ‘program’ and ‘health care’, among others. Search results were downloaded or copied to an Excel document; if that was not possible, the reviewer conducted stage 1 screening (see Section 2.4) by reviewing titles and document descriptions and downloaded or copied the records retained for stage 2 screening.

### 2.3 Eligibility criteria

To be eligible for inclusion, articles had to describe a family‐centred care intervention for children 12 years and younger with chronic conditions and/or their families (Table 1). Operationalization of these concepts is described below. These criteria were designed to balance a desire to include a wide range of recently published family‐centred care interventions.

**Table 1:** Eligibility criteria.

	Inclusion	Exclusion
Study design	Primary studies of any design except those specifically excluded Clinical practice guidelines Theoretical articles (must describe family‐centred care model or framework AND indicate it was peer‐reviewed)	Systematic reviews and other secondary review studies Case series Primary studies with <10 participants, including case studies
Population	Children (aged ≤12 years) with chronic conditions and/or their families (target of the intervention and study, or study alone)	Interventions or studies targeting solely adolescents (aged 13–18 years)
	Articles where the age or the health care needs of the study population are not clear but possibly eligible will be included	Health care needs are related to short‐term disability or acute injury (reasonably expected to last <1 year); neonatal or infant conditions expected to last <1 year
	If <100% are aged ≤12 years, include if:	
	*Primary studies*: ≥50% are ≤12 years *Clinical guidelines*: Article clearly specifies intervention is targeted at children	
	If <100% are children with ongoing, elevated health care needs, include if:	
	50% are children with ongoing needs	
Concept	Family‐centred care interventions	Prevention or screening interventions for a condition if the population does not already have a condition requiring ongoing, elevated health care
	The article, or a cited document, must clearly describe all of the following:	Clinical drug treatment regimens
	That a main purpose of the intervention is to improve the population's experiences with health care The intervention's activities or processes That both (a) health care providers and/or administrators and (b) primary caregivers (and children or other family members) were actively engaged in either the development or research of the intervention	Interventions targeting transition from paediatric to adult services
Context	All settings, all health care professionals involved included	N/A
Outcomes	All outcomes	N/A
Publication date	Published in or after 2019	N/A
Language	English	Anything other than English
Publication type	Published, full‐length articles or grey literature reports, policy documents	Unpublished articles, books Protocols, conference abstracts, theses or dissertations, commentaries and letters

Abbreviation: N/A, not available.

#### 2.3.1 Study design types

Primary studies of any design were eligible except for case series and primary study designs with fewer than 10 participants, including case studies. Theoretical articles describing family‐centred care models or frameworks that have not yet been implemented were included if the article had been peer‐reviewed. Clinical practice guidelines were also included. Systematic and other secondary review studies were excluded as we anticipated that they would be repetitive.

#### 2.3.2 Population

We included studies where the target of the intervention was children aged 12 years or younger with chronic conditions and/or their families. Interventions or studies that targeted solely adolescents (aged 13–18 years) were excluded as their health care needs are often distinct from those of younger children. ‘Chronic conditions’ were defined as requiring an elevated number or intensity of interactions with the health care system relative to the general paediatric population, expected to be required for 1 year or more.

#### 2.3.3 Concept

We included articles that described family‐centred care interventions (activities, strategies or policies).^38^ Family‐centred care was defined as an approach to the planning, delivery and evaluation of health care that was grounded in mutually beneficial partnerships among health care providers, patients and families.^13^ We considered an intervention to be family‐centred if: the main objective of the intervention was to improve health care experiences (including timely access to care or treatment, coordination of care, communication with providers and emotional support) for the population; and both (i) health care providers and/or administrators and (ii) primary caregivers of children were actively engaged in the development or research of the intervention (e.g., in the study of implementation of the intervention). With respect to this latter criterion, we considered engagement of primary caregivers and providers/administrators to be critical given their roles as knowledge users positioned to benefit from or to implement respectively, interventions to improve family‐centred care. Included articles had to describe the intervention's activities or processes or cite other articles accessible to the authors that did. We excluded: screening or preventive interventions targeting children that did not have a pre‐existing, chronic condition; clinical drug treatment regimens, which are typically not generalizable and do not directly target improvement of health care experiences; interventions targeting transition from paediatric to adult health care services; institutional networks and partnerships; and existing services (longstanding, geographically widespread programmes that were not new interventions to improve care).

#### 2.3.4 Context

We included interventions that addressed physical or mental health care for a child, were implemented in any setting (e.g., health care, school, home) for acute or nonacute care and involved any health care professional (e.g., physician, nurse, rehabilitation therapist).

#### 2.3.5 Other

We excluded protocols, conference abstracts, theses and dissertations, commentaries and letters. We included articles irrespective of the outcomes studied. Only articles published in or after 2019 were included. As many grey literature websites and documents did not report a publication date, we chose to include all undated grey literature. Only articles published in English were included.

### 2.4 Study selection

In stage 1 of a two‐stage screening process, we used a liberal accelerated approach.^39^ Two reviewers (among A. J. C., Z. A., R. I.) independently screened the titles and abstracts of identified records. A record was retained if at least one reviewer positively assessed that it met the eligibility criteria or was uncertain about its inclusion. For the grey literature, one author (A. J. C. or Ammar Saad) performed stage 1 screening.

In stage 2, the full‐text articles of all records retained in stage 1 were obtained. If an article could not be obtained electronically free of charge, we contacted the corresponding or lead study author by email up to two times and excluded the article if a copy could not be obtained. Two reviewers (among A. J. C., Z. A., R. I., Ammar Saad) independently screened all full‐text articles and grey literature in duplicate. We resolved disagreements by discussion and consensus; an arbitrator (B. K. P.) resolved disagreements that could not be resolved by the reviewers. Clinical experts (P. C., M. K.) provided input if the health care needs criterion could not be determined by the reviewers. If a full‐text article and its cited references did not report enough information to decide eligibility (e.g., regarding the population targeted by an intervention), we excluded the article. We did not contact study authors to request additional information.

### 2.5 Data charting

Key information from included articles was charted using verbatim information into a standardized, predefined, piloted Excel data charting form (Appendix S3) by one person (among Ammar Saad, Z. M., A. H., Z. A.), then verified by a second, different study team member (among A. J. C., Ammar Saad, R. I.). If multiple articles described the same intervention, we charted the information from each article separately and collated them during synthesis. If an article referenced a previous article published before 2019, we charted from the previous article only information related to the intervention.

We charted four main types of information: (1) the characteristics of each article (lead author, title, journal, year of publication); (2) study participant types for primary studies (e.g., children, family members, health care professionals), when applicable; (3) details about the development of each intervention (i.e., activities or processes used; engagement of groups such as patients, caregivers and health care providers) and characteristics of the children targeted by the intervention (i.e., age range, type of health care need); and (4) using an adapted version of the Template for Intervention Description and Replication (TIDieR) checklist.^40^ We charted information about the reporting of each intervention including: the care delivery setting involved, the authors' stated objectives and rationale for, or theory grounding, each intervention, and the intervention's components (specific activities/processes). Our adapted checklist did not include TIDieR checklist items #11 and #12, about planned and actual fidelity to the intervention, as we did not aim to assess intervention effectiveness.

### 2.6 Synthesis

Using the data charted, we synthesized the development of the interventions by activity type (e.g., feedback seeking, pilot testing) and groups engaged in development (e.g., caregivers, children, health care providers). We also summarized the broad care delivery settings (i.e., inpatient, outpatient, community) and the chronic conditions of the target populations, grouping by pathophysiological manifestations and symptoms.

We summarized the activities/processes and objectives of each intervention and identified which among eight family‐centred care domains the intervention sought to address^21^, ^22^ (definitions, Appendix S4). For the latter characterization, we used an iterative coding approach, where one reviewer (Ammar Saad) coded and a second team member (A. J. C.) verified. We summarized the common types of intervention approaches within each family‐centred care domain.

## 3 RESULTS

### 3.1 Study selection

**Figure 1:**
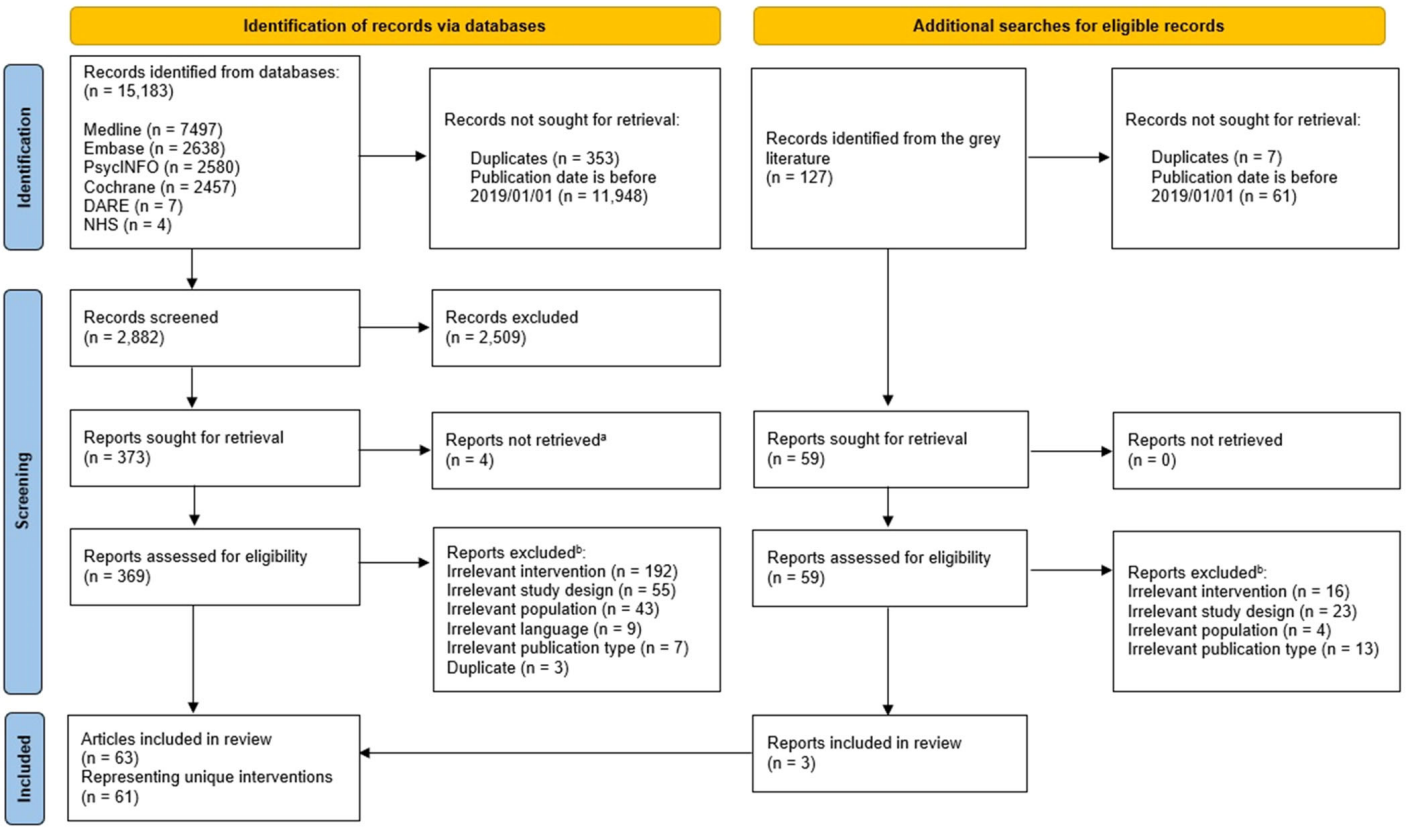
Preferred Reporting Items for Systematic reviews and Meta‐Analyses flow diagram of study screening and selection.

After deduplication and removal of records published before 2019, we screened 2882 records by their titles and abstracts; of those, 369 full‐text reports were assessed for eligibility (Figure 1). We further identified 127 records through grey literature searches, and after deduplication and removal of records published before 2019, we retrieved and assessed 59 full‐text reports for eligibility (Figure 1). A total of 63 reports were included in this review, representing 61 unique interventions.^41^, ^42^, ^43^, ^44^, ^45^, ^46^, ^47^, ^48^, ^49^, ^50^, ^51^, ^52^, ^53^, ^54^, ^55^, ^56^, ^57^, ^58^, ^59^, ^60^, ^61^, ^62^, ^63^, ^64^, ^65^, ^66^, ^67^, ^68^, ^69^, ^70^, ^71^, ^72^, ^73^, ^74^, ^75^, ^76^, ^77^, ^78^, ^79^, ^80^, ^81^, ^82^, ^83^, ^84^, ^85^, ^86^, ^87^, ^88^, ^89^, ^90^, ^91^, ^92^, ^93^, ^94^, ^95^, ^96^, ^97^, ^98^, ^99^, ^100^, ^101^, ^102^, ^103^ Furthermore, we consulted additional publications, which were cited in the eligible reports, for additional details about 23 of the 61 included interventions during our data charting.^104^, ^105^, ^106^, ^107^, ^108^, ^109^, ^110^, ^111^, ^112^, ^113^, ^114^, ^115^, ^116^, ^117^, ^118^, ^119^, ^120^, ^121^, ^122^, ^123^, ^124^, ^125^, ^126^, ^127^, ^128^, ^129^, ^130^, ^131^, ^132^

### 3.2 Study characteristics

Table 2 and Appendix S5 describe the 63 included articles, the majority of which reported on primary research studies (*n* = 57; 90.5%). The most common primary study design was quasiexperimental (*n* = 18; 28.6%), followed by randomized controlled trial (RCT) (*n* = 11; 17.5%). The sample size of the 57 included primary research studies ranged from 10 (per our inclusion criteria) to 6,259 participants, with almost a third reporting a sample size of 10–30 participants (*n* = 18/57; 31.6%). Seventy‐nine percent of articles described studies conducted in (or, if not stated, country of affiliation of lead author) North America (*n* = 40; 63.5%), as well as Europe (*n* = 6; 9.5%) and the United Kingdom (*n* = 4; 6.3%).

**Table 2:** Characteristics of included articles.

Study characteristics	*N* = 63, *n* (%)
Study design	
Primary studies	57 (90.5)
Quasi‐experimental studies	18 (28.6)
Randomized controlled trials	13 (20.6)
Mixed methods studies	10 (15.9)
Qualitative studies	9 (14.3)
Cohort studies	4 (6.3)
Cross‐sectional studies	3 (4.8)
Clinical guidelines	3 (4.8)
Theoretical articles	3 (4.8)
Sample size^a^	
10–30	18 (28.6)
31–90	17 (27.0)
91–300	14 (22.2)
>300	7 (11.1)
Not reported	1 (1.6)
Not applicable	6 (9.5)
Geographic location	
North America	40 (63.5)
South/East Asia	7 (11.1)
Europe	6 (9.5)
United Kingdom	4 (6.3)
Australia	1 (1.6)
South America	1 (1.6)
Africa	1 (1.6)
Unspecified (clinical guidelines)	3 (4.8)
Health care conditions	
Multisystem, life‐limiting or complex conditions	14 (22.2)
Developmental conditions and delays	10 (15.9)
Pulmonary conditions	9 (14.3)
Mental and behavioural conditions	5 (7.9)
Oncological conditions	4 (6.3)
Physical and motor disabilities	4 (6.3)
Cardiological conditions	3 (4.8)
Haematological conditions	3 (4.8)
Neurological and cognitive conditions	3 (4.8)
Autoimmune disorders	2 (3.2)
Endocrinological conditions	2 (3.2)
Renal conditions	1 (1.6)
Uncategorized	3 (4.8)

^a^ Primary studies only.

### 3.3 Intervention development, including which groups were engaged in development

**Figure 2:**
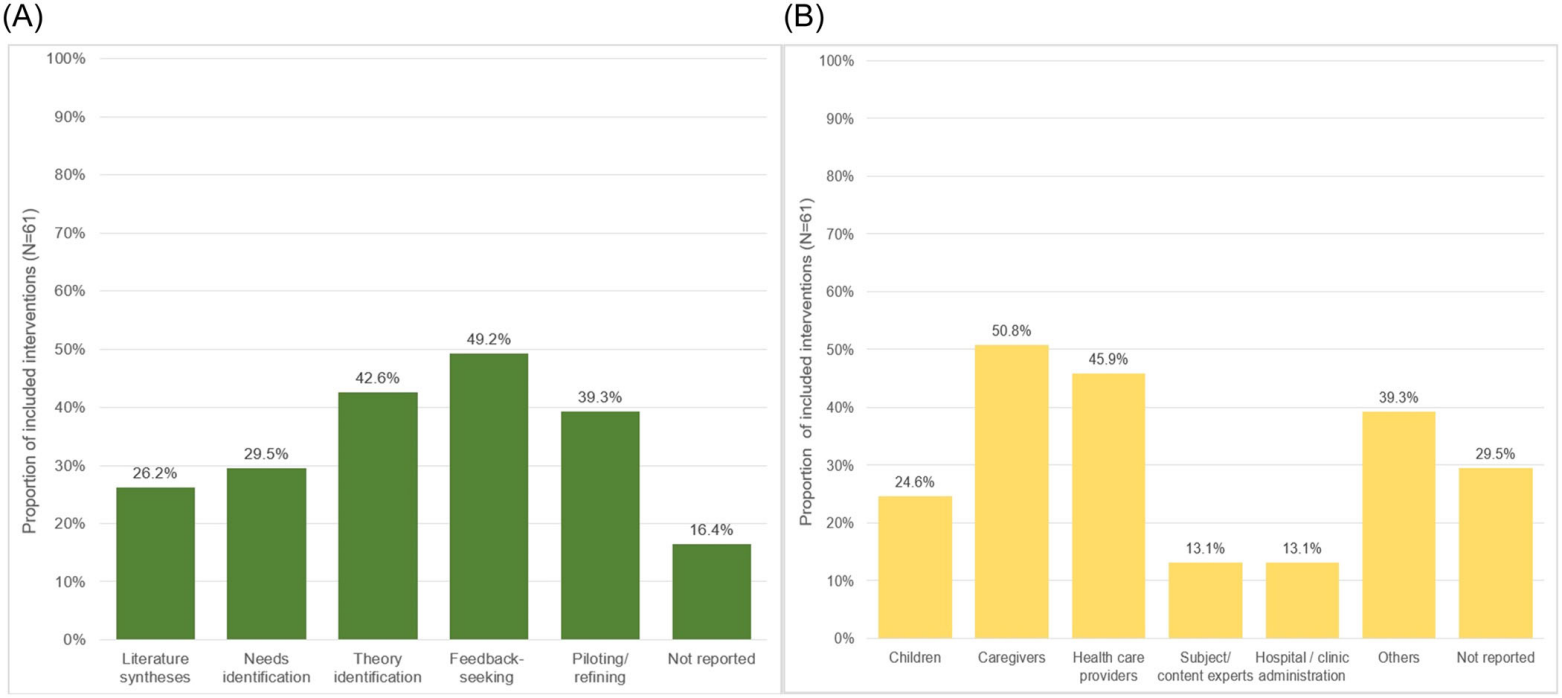
(A) Activities involved in intervention development. Feedback‐seeking activities: Quantitative and qualitative data collection of opinions and feedback from contributor groups on content or design. Piloting/refining: Feasibility testing and pilot implementation of interventions. (B) People and entities engaged in intervention development alongside researchers. Caregivers: Parents, caretakers and legal authority representatives. Subject/content: Clinical or intervention design expertise. Others: Allied health professionals, information technology specialists, insurers, teachers, nongovernmental organizations, community advocates and governmental health entities.

For the majority of the 61 interventions, authors provided or cited information about the activities or processes involved in intervention development (*n* = 51; 83.6%) and the groups engaged in their development (*n* = 43; 70.5%) (Figure 2A,B). Common activities included the elicitation of feedback from patients, caregivers or health care providers on intervention content or design (*n* = 30), the use of intervention theories or frameworks in conceptualization (*n* = 26) and piloting/refining interventions with end users (*n* = 24; Figure 2A and Appendix S5). Among interventions where authors described who was involved in development, caregivers (e.g., parents) were engaged in developing about half the interventions (*n* = 31; Figure 2B and Appendix S5). A similar proportion described involving health care providers (*n* = 28), whereas child patient involvement was described in the development of 15 interventions.

### 3.4 Intervention characteristics

Among the 63 articles, there were 61 unique interventions described (Table 3). Most interventions aimed to improve care experiences in one setting: outpatient (*n* = 22; 36.1%), inpatient (including emergency department) (*n* = 10; 16.4%) or community (*n* = 9; 14.8%) settings. A smaller number of interventions addressed shared or transitional care between outpatient and community (*n* = 11; 18.0%), inpatient and community (*n* = 7; 11.5%) or inpatient and outpatient settings (*n* = 2; 3.3%).

**Table 3:** Characteristics of included interventions.

References	Intervention description	Intended recipients	Timing (T) and duration (D)	Delivery mode (M) and format (F)
	Interventions delivered in or from outpatient clinical settings (*n* = 22)			
[41]	Website to prepare parents to take part in decision‐making during their consultation with a rehabilitation physician; helps families identify their needs + access information	Caregivers	T: As desired D: Not applicable	M: Virtual F: Individual
[42, 43]	Collaborative meetings between families + therapists, at clinic or home (family preference), to codevelop + implement child development intervention, including family needs, activities + goals	Family, HCPs	T: Six weekly sessions D: Approximately 40 min each	M: IP F: Individual
[44]	Disease‐specific medical home care coordination programme that includes caregiver education (guidebook), team‐based care + clinical care plan	Children, caregivers	T: U/S D: U/S	M: U/S F: U/S
[46]	Online tool to engage caregivers in care planning. Caregivers identify + prioritize activities for participation and identify goals, attainment strategies, possible supports + barriers which are incorporated into a care plan report	Caregivers	T: U/S D: U/S	M: Virtual F: Individual
[49]	Online platform that: provides caregivers with information + resources on rehabilitation; facilitates communication (chat, video conference) with therapists + includes a forum for discussions among caregivers or with HCPs	Caregivers	T: U/S D: U/S	M: Virtual F: Individual
[56]	Educational video for caregivers about symptoms, treatment + side effects of leukaemia + testimonials, support from other families	Caregivers	T: Watched when timely D: 23 min	M: Virtual F: Individual
[58]	Tools + aids that HCPs use to facilitate communication with caregivers about care, family life + support needs; tool for caregivers to indicate care preferences	Children, caregivers, HCPs	T: U/S D: U/S	M: U/S F: U/S
[64]	Gamified, interactive digital communication tool for child use in waiting rooms to highlight their symptoms, worries + fears to the HCP	Children, HCPs	T: U/S D: U/S	M: IP; virtual F: U/S
[66]	Palliative care/end of life communication sessions incorporated into clinic visits to plan treatment, discuss prognosis, encourage caregiver articulation of goals + values + support caregivers emotionally	Caregivers	T: Three sessions, 4–26 weeks after assessment D: Depends^a^	M: IP F: U/S
[69]	Collection of patient‐reported outcomes, collaboratively identified by families + HCPs, during routine care, followed by co‐development of personalized future care plans	Children, family, HCPs	T: Access 1–2×/week D: 1 week to 8 months	M: Virtual F: Individual
[71]	Paediatric rehabilitation coaching session in which child + family's views, values and needs are identified + used to individualize a care plan	Children, caregivers, family	T: U/S D: U/S	M: U/S F: U/S
[72]	Redesign of the clinic's booking system to give caregivers responsibility to book routine consultations; provides information to caregivers on ideal consultation frequency, advice on when + how to seek help as needed	Children, caregivers	Not applicable	M: IP; virtual F: Individual
[77]	Tailored, structured decision coaching sessions to help families identify treatment preferences, with emphasis on child input before caregiver input	Children, caregivers	T: U/S D: U/S	M: IP F: Individual
[78]	A paper‐based tool to facilitate documentation + discussion of care plan specifics (i.e., actions, people responsible, timeline), completed by families with help from nurse practitioner	Children, family, HCPs	T: 1 FU 2–3 weeks after first meeting D: First meeting 2 h, FU depends^a^	M: IP; virtual F: Individual
[82]	Care plan, developed by HCPs, is integrated into patient's electronic health record + made accessible via an online patient portal to facilitate communication between medical team + caregivers	Caregivers, HCPs	T: U/S D: Not applicable	M: Virtual F: Individual
[89]	Virtual platform to improve communication between families + HCPs: access to an individualized care plan, goal setting facilitation, medical history, educational resources. Families decide who may access sensitive information	Children, caregivers, HCPs	Not applicable	M: Virtual F: Individual
[90]	Educational programme includes assessing information needs using a checklist before a tailored physician‐led education session (with booklet) on disease, treatment + complications	Children, caregivers	T: 1 FU 4 weeks after first session D: First contact 20–30 min; FU U/S	M: IP F: U/S
[92]	Online portal for families to log asthma symptoms, facilitating self‐monitoring + education and clinic awareness of changes, patient monitoring + timely follow‐up	Children, caregivers, HCPs	T: Weekly D: 1 year	M: Virtual F: Individual
[94]	Educational programme to improve HCP management of asthma, cross‐cultural communication + education practices via lectures, case studies, videos + interactive discussions	HCPs	T: Two sessions over 2 weeks D: 2.5 h each	M: IP F: Group
[97]	To encourage child involvement in clinic visits, waiting room educational videos delivered via iPad + question prompt list covering questions that the child/family may want to ask the HCP	Children, caregivers	T: One video session; prompt list U/S D: Videos 11 min (1–2 min each); prompt list U/S	M: IP F: Individual
[102]	Secure online system for communication among providers (so that caregivers do not have to act as messengers) + between providers + caregivers (communications shared with the child's care network)	Caregivers, HCPs	T: Depends^a^ D: 6 months total	M: Virtual F: Individual
[103]	A communication “passport” tool used by children + caregivers to track details of services received + outcomes; information is shared with other HCPs	Children, caregivers	Not applicable	M: IP F: Individual
	Interventions delivered in or from inpatient clinical settings and emergency departments (*n* = 10)			
[45]	Recommendations for hospital cost discussions with caregivers: where (at bedside), when (when child's medical status is stable) + how (should be transparent, optional + tailored to families' needs + preferences)	Caregivers	T: U/S D: U/S	M: IP F: U/S
[47]	Co‐development of early palliative care plan; support for caregivers to care for child's needs in the NICU; emotional + psychological support for caregivers + help to create visual memories	Children, siblings, caregivers	T: Depends^a^ D: U/S	M: U/S F: U/S
[53]	Perioperative programme responsive to the individual needs of children with ASD. All stages of surgery, from preparation to discharge, are designed to manage stress + provide comfort	Children, caregivers	T: U/S D: Varied: presurgery to 1 day postsurgery	M: IP; virtual F: U/S
[61]	NICU physical space redesign. Newborns are admitted to six‐bed pods + placed in single‐family rooms when they require stepped‐down care; family spaces accommodate family needs (e.g., showers + overnight stays)	Children, caregivers	T: 24/7 access, caregiver meetings 1×/week D: Not applicable	M: IP F: Individual
[75]	Cardiac surgery preparation to alleviate stress + anxiety in child patients through videos, games + toys + in caregivers through education + counselling	Children, caregivers	T: Once before surgery D: U/S	M: IP F: U/S
[76]	Mediation session with a transcultural team that facilitates: linguistic + cultural translation of medical information; families' articulation of their understanding + views of treatment options; collaboration between family + HCPs on a common story about the child's disease + life	Children, family, HCPs	T: One session D: 2–4 h	M: IP F: Individual
[79]	Enhancement of usual care with complementary therapies to alleviate pain + anxiety; therapies selected + tailored through collaboration between the child, family, nurse + integrative medicine specialist	Children, caregivers	T: Depends^a^ D: Depends^a^	M: IP F: Individual
[81]	An animated robotic toy to provide medical play, offer distraction + facilitate emotional expression by children with cancer at the hospital or home	Children	T: Depends^a^ D: 3 days	M: IP F: Individual
[96]	Asthma care improvement initiative in the emergency department: processes to administer medication at admission + discharge; discharge instructions, care plan; team‐based communication; Spanish translation	Children, caregivers	T: One session predischarge D: 8 min	M: IP F: U/S
[99]	Two‐phase programme to support mothers by engaging them in safe infant care in the NICU + during transition to the general ward, providing information + negotiating mother participation preferences during NICU visiting hours	Caregivers	T: 2×/day D: Phase 30 min each; total duration depends^a^	M: IP F: Individual
	Interventions to improve experiences of care delivered in the community (e.g., home, school, community clinic) (*n* = 9)			
[48]	Primary care physicians can access rapid consultations with a child clinician/psychiatrist to answer questions+/or initiate referrals	HCPs	T: U/S D: U/S	M: IP; virtual F: U/S
[51]	HCPs + families co‐develop an action care plan; an online patient portal facilitates care follow‐up and family‐provider communication	Children, family	T: U/S D: U/S	M: IP; virtual F: U/S
[52]	School‐based psychiatric service to increase access: free assessment, treatment recommendations + follow‐up care support (e.g., wrap‐around needs, appropriate transition to community‐based mental health services)	Children, caregivers	T: Depends.^a^ 3–4 FU D: U/S	M: IP F: U/S
[59]	Community‐based, ‘one‐stop‐shop’ allergy clinic; a general practitioner with allergy specialization, dietitian + nurse provide assessment, care coordination with primary care + care advice	Children	T: Depends^a^ D: First assessment 30 min, FU depends^a^	M: IP F: U/S
[65]	Strategies to improve access to a school‐based asthma programme: (1) allow treatment to proceed with verbal caregiver consent; (2) give asthma care plan to caregivers; (3) support school staff to screen for potential cases	Children, caregivers	Not applicable	M: IP F: U/S
[67]	Early childhood, home‐based intervention; involves family needs assessment, individualizing a care plan, five home visits to coach caregivers on behavioural management skills + support to implement learned skills	Children, caregivers, family	T: Five home visits D: 1–1.5 h each over 3 months	M: IP; virtual F: Individual
[83]	Team‐based, social worker‐led community care coordination, with regularly updated care coordination binder (care plan, medications, contact information)	Children	T: ≥1×/3 months D: Initial 1 h, FU 40 min each	M: IP; virtual F: Individual
[84]	Child‐centred care model that includes capacity‐building/training for health care workers, age‐appropriate education tools for children + child‐friendly spaces in clinics	Children, caregivers, HCPs	Not applicable	M: IP F: U/S
[91]	Multidisciplinary team supports community care for children with neurodevelopmental disorders; educates + supports caregivers + preschool staff; delivers individualized developmental programmes for children	Children, caregivers	T: Biweekly, number depends^a^ D: U/S	M: IP F: U/S
	Interventions shared or transitioned between outpatient clinical settings and the patient's community (*n* = 11)			
[54]	Training for caregivers on managing their child's conduct problems at home (educational videos followed by virtual sessions with a clinician)	Caregivers	T: Videos before sessions; 6–10 weekly sessions D: Videos 7–19 min each, sessions 50–60 min each	M: Virtual F: U/S
[55]	Telehealth consultation with a surgeon/specialist for remote families; includes education + coordination of lab testing	Children, family	T: U/S D: 30–45 min each	M: Virtual F: Individual
[62]	Epilepsy telemedicine programme connecting rural posts to a secondary care centre to facilitate tertiary referral, ensure follow‐up + improve consistent access to antileptics	Children, caregivers	Not applicable	M: Virtual F: Individual
[68]	Telemedicine intervention connecting rural families from a remote clinic office to haematologist + other allied health professionals; on‐site physical exams	Children, caregivers	Not applicable	M: IP; virtual F: Individual
[73, 74]	Collaborative management of health condition; coordinated by a care manager + primary care providers. After identification of family goals, coordination of on‐site services with specialists: education, training, support + coaching for caregivers on home management	Children, caregivers, HCPs	T: Six to 12 sessions D: Up to 12 h over 6 months	M: IP; virtual F: Individual; group
[85]	Group psychoeducation programme to improve children's knowledge of their medications + help caregivers support their children to take on a larger role in managing their medications	Children, caregivers	T: Five sessions D: 90 min each	M: IP F: Group
[86]	CPG recommends, inter alia: sharing information tailored to child capacity, family needs + preferences; development of care plan for caregivers' needs; access to trained advocate; HCP cultural competency training	Children, caregivers, HCPs	T: U/S D: U/S	M: U/S F: U/S
[87]	CPG recommends, *inter alia:* creating a shared treatment plan; tailoring information + support for children + families; identifying child concerns + preferences	Children, caregivers, HCPs	T: U/S D: U/S	M: U/S F: U/S
[95]	Care coordination by paediatric hospitals to enable children with medical complexity to remain with a local medical home + local services; includes home visit to assess needs + barriers to care, team review of the case, development of care plan + coordination strategy	Children, caregivers, HCPs	T: Home visit, team review: 1 each, 2 FU 1 + 6 months after D: 45–120 min, FU U/S	M: IP; virtual F: Individual
[98]	Telemedicine consultation connecting children + families at home to a renal dietitian to facilitate information sharing about diagnosis + treatment	Children, family	T: U/S D: U/S	M: Virtual F: Individual
[100]	Single point of access to mental health services. Children are assessed after referral + assigned to suitable providers. Care + recovery are tailored to child's needs. Programme includes crisis team + after‐hour services	Children, caregivers	T: U/S D: U/S	M: IP; virtual F: U/S
	Interventions shared or transitioned between inpatient clinical settings and the patient's community (*n* = 7)			
[50]	Online care coordination to reduce system expenditures; includes: development of a care plan, advice on health systems navigation, access to a range of services, health education + consultations/coaching with community health workers	Children, caregivers	T: First meeting + FUs every 2–12 weeks; Depends^a^ D: U/S	M: IP; virtual F: U/S
[60]	Medical home programme following NICU discharge; predischarge needs assessment + home care education; post‐discharge primary care service, support to access care and services, emotional support + structured team care coordination	Children, caregivers	T: Depends^a^ D: First meeting 30–60 min, FU U/S	M: IP; virtual F: Individual
[63]	A nurse provides support for home care, including a 6‐month care plan, home management education, support after discharge + online platform for timely problem‐solving	Children, family	T: Visits U/S, phone 1×/week D: 6 months	M: IP; virtual F: Individual
[70]	Early neurorehabilitation programme in which a multidisciplinary specialist team conducts needs assessment, coordinates with community care, proactively plans discharge + supports transition to long‐term or local care	Children	T: U/S D: Over course of 24 h	M: IP F: U/S
[80]	Placement of a paediatric trauma nurse to coordinate care for high risk inpatients. Follows care from admission to postdischarge, coordinates follow‐up care services, liaises with child's care team, communicates with the family	Children, family, HCPs	T: Depends^a^ D: Underreported (≥3 months)	M: IP; virtual F: Individual
[88]	CPG recommends, inter alia: involving a multidisciplinary team in addressing care needs + developing family‐centred communication strategies	Children, caregivers, HCPs	T: U/S D: U/S	M: U/S F: U/S
[93]	Hospital‐to‐home transition programme ensures families receive medication before discharge, coordinates with primary care and schools, provides telephone access to a patient navigator postdischarge + makes a referral for a home evaluation of asthma triggers	Children, caregivers, HCPs	T: Navigator contact 1–2/month; coordination, home evaluation U/S D: Over 6 months	M: IP; virtual F: Individual
	Interventions shared or transitioned between inpatient and outpatient clinical settings (*n* = 2)			
[57]	Care coordination by designated providers: Nurse is a member of the care team; provides continuity during hospitalization, accompanies families to outpatient visits, maintains care plan. Care coordination assistant provides logistical + emotional support to families	Children, caregivers	Not applicable	M: IP; virtual F: U/S
[101]	Group programme to educate children with chronic pain and their caregivers about pain management strategies (relaxation, cognitive behavioural skills) through a video, workbook + goal‐setting exercises	Children, caregivers	T: One session D: Session U/S, video 40 min	M: IP F: Individual; group

Abbreviations: CPG, clinical practice guideline; FU, follow‐up; HCP, health care provider; IP, in‐person; NICU, newborn intensive care unit; U/S, unspecified.

^a^ ‘Depends’ defined as: Intervention timing and/or duration depends on the needs and preferences of the recipients and/or their families

We completed TIDieR checklists for each intervention (Appendix S6). This review's eligibility criteria for interventions were broader than what the TIDieR checklists were intended to address. Three included interventions were clinical practice guidelines^86^, ^88^, ^122^ and one was a set of recommendations^45^; we did not complete TIDieR checklists for these interventions. The quality of reporting for the remaining 57 included interventions varied: 10 interventions reported eight or nine TIDieR checklist items in sufficient detail, 26 interventions reported six or seven items, and 21 reported four or five items (Appendix S7). The checklist items least often reported were: whether the intervention was modified during the study (item #10, *n* = 2), whether the intervention was planned to be tailored (item #9, *n* = 22), the number of times the intervention was delivered and over what period (item #8, *n* = 44), and what materials were used in the intervention (item #3, *n* = 45) (Appendix S7).

Interventions targeted children (*n* = 48; 78.7%), their caregivers (*n* = 54; 88.6%), and health care providers (*n* = 20; 32.8%), with several interventions targeting multiple groups. Ten interventions (16.6%) specified ‘the family’ as a targeted recipient group without specifying further. One intervention (1.6%) targeted siblings.^47^ Interventions were delivered in‐person (*n* = 23; 37.7%), virtually (*n* = 13; 21.3%) or using both modes (*n* = 18; 29.5%). Seven interventions (11.5%) did not specify a delivery mode.

### 3.5 Family‐centred care domains addressed by interventions and intervention components

**Figure 3:**
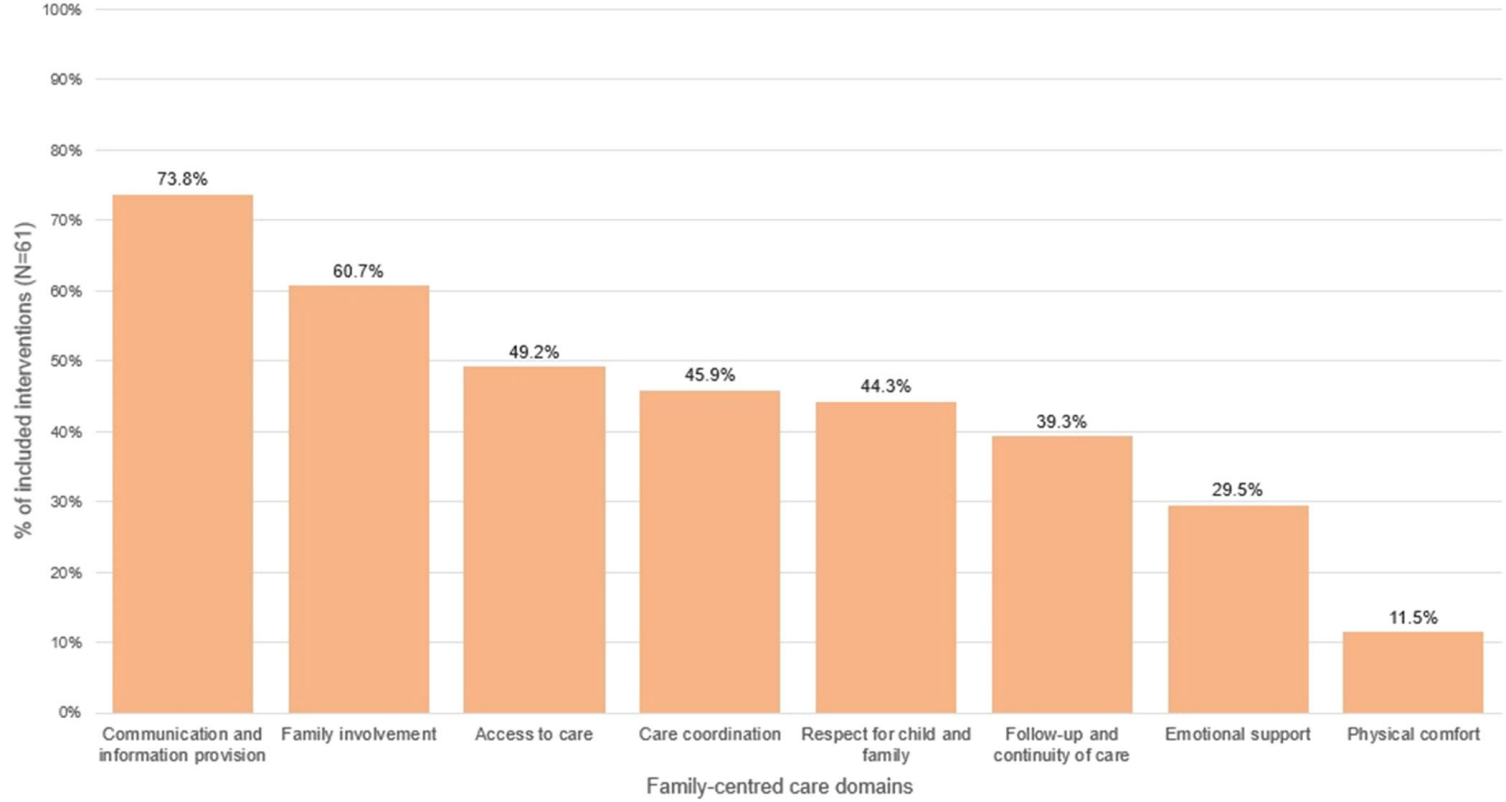
Proportion of interventions addressing selected family‐centred care domains.

Most interventions (*n* = 56; 91.8%) addressed more than one of the eight family‐centred care domains that we considered, with nearly half (*n* = 26, 42.6%) addressing at least four domains (Appendix S8). The most common domains addressed by interventions were communication and information provision (*n* = 45; 73.8%), family involvement (*n* = 37; 60.7%) and access to care (*n* = 30; 49.2%) (Figure 3). The least common domains addressed by interventions were physical comfort (*n* = 7; 11.5%) and emotional support (*n* = 18; 29.5%) (Figure 3).

Table 4 describes types of approaches taken to addressing each family‐centred care domain, with intervention examples. For example, care coordination was most commonly addressed by adopting a team‐based approach to care delivery, in which services are provided by a multidisciplinary team in the patient's medical home or via a single point‐of‐care (15/28 interventions). We identified more types of intervention approaches to the access to care domain (*n* = 6) than any other domain.

**Table 4:** Approaches to addressing family‐centred care domains.

Approaches	Number of interventions	Implementation examples
Communication and information provision	45^41^, ^42^, ^43^, ^44^, ^45^, ^49^, ^50^, ^51^, ^52^, ^53^, ^54^, ^56^, ^57^, ^58^, ^60^, ^61^, ^63^, ^64^, ^66^, ^67^, ^73^, ^74^, ^75^, ^76^, ^77^, ^78^, ^80^, ^82^, ^84^, ^85^, ^86^, ^87^, ^88^, ^89^, ^90^, ^91^, ^92^, ^93^, ^94^, ^96^, ^97^, ^98^, ^99^, ^100^, ^101^, ^102^, ^103^	
In‐person interactions with providers	27^42^, ^43^, ^44^, ^45^, ^51^, ^52^, ^53^, ^57^, ^60^, ^61^, ^63^, ^66^, ^67^, ^73^, ^74^, ^75^, ^76^, ^77^, ^80^, ^85^, ^86^, ^87^, ^88^, ^90^, ^91^, ^94^, ^96^, ^99^, ^101^	Educational sessions to assess and address families' information needs Training sessions to coach children and their families on how to manage the disease
Tools and aids	27^41^, ^42^, ^43^, ^44^, ^49^, ^50^, ^51^, ^54^, ^56^, ^58^, ^63^, ^64^, ^66^, ^75^, ^78^, ^82^, ^84^, ^86^, ^89^, ^90^, ^92^, ^94^, ^96^, ^97^, ^99^, ^101^, ^102^, ^103^	Educational aids (videos, printed materials) providing caregivers with information about the disease, symptoms and management Communication tools (online platforms, mobile apps, checklists) facilitating bidirectional information sharing with providers
Using technology to facilitate information sharing	9^49^, ^53^, ^54^, ^55^, ^89^, ^93^, ^98^, ^100^, ^102^	Telemedicine programmes to educate and train caregivers on how to manage symptoms
Family involvement	37^41^, ^42^, ^43^, ^45^, ^46^, ^47^, ^50^, ^51^, ^53^, ^54^, ^57^, ^58^, ^60^, ^61^, ^66^, ^67^, ^69^, ^71^, ^73^, ^74^, ^75^, ^76^, ^77^, ^78^, ^79^, ^82^, ^85^, ^86^, ^87^, ^88^, ^89^, ^90^, ^91^, ^92^, ^95^, ^97^, ^99^, ^100^, ^101^	
Involving families in decision‐making about care or treatment	22^41^, ^42^, ^43^, ^45^, ^46^, ^51^, ^57^, ^58^, ^66^, ^67^, ^69^, ^71^, ^73^, ^74^, ^76^, ^77^, ^78^, ^79^, ^82^, ^88^, ^89^, ^90^, ^95^, ^100^	Provide decision‐making tools (e.g., checklists, mobile applications) Adaptation of a decision‐making process (e.g., decision‐making discussions) Train families to be involved in decision‐making
Involving families as recipients of care and focusing on their well‐being	13^47^, ^50^, ^53^, ^54^, ^57^, ^58^, ^60^, ^66^, ^73^, ^74^, ^75^, ^86^, ^87^, ^88^	Assess family members' wellbeing and include their needs in the care plan Train family members to self‐regulate their emotions Support family members emotionally and psychologically during and outside clinical visits
Supporting families to participate in the care of the child	14^42^, ^43^, ^47^, ^53^, ^54^, ^60^, ^61^, ^67^, ^71^, ^85^, ^91^, ^92^, ^97^, ^99^, ^101^	Train family members to provide care to the child (e.g., day‐to‐day support) Identify family members' roles and responsibilities within the care plan Ensure timely family presence during care (i.e., during hospitalization)
Access to care	30^44^, ^48^, ^49^, ^50^, ^51^, ^52^, ^53^, ^54^, ^55^, ^57^, ^58^, ^59^, ^60^, ^62^, ^63^, ^65^, ^72^, ^73^, ^74^, ^83^, ^86^, ^88^, ^89^, ^91^, ^93^, ^95^, ^96^, ^98^, ^100^, ^101^, ^103^	
Using technology to connect families to providers	12^49^, ^53^, ^54^, ^55^, ^57^, ^72^, ^73^, ^74^, ^83^, ^88^, ^89^, ^93^, ^98^	Telemedicine‐based programme connecting remote families to providers Access to providers via telephone or email channels to receive support
Programme or care delivery in communities	10^48^, ^52^, ^59^, ^60^, ^65^, ^88^, ^91^, ^95^, ^100^, ^101^	School‐based programme Medical home approach to care delivery in the community Home visits
Facilitating timely and affordable linkage to hospital or community services	8^44^, ^50^, ^52^, ^53^, ^58^, ^60^, ^72^, ^103^	Checklists to assess service needs Booking system accessible to caregivers Designate community health workers to deliver care
Appointment scheduling and logistic support	6^52^, ^57^, ^60^, ^72^, ^83^, ^93^	Transportation support Designate a team member to schedule appointments
Improving availability of and access to medications	6^57^, ^62^, ^73^, ^74^, ^88^, ^93^, ^96^	Medication supply to rural posts (connected via telemedicine) Dispense medications at discharge
Access to an online platform for treatment and support	4^49^, ^50^, ^51^, ^63^	Websites or mobile applications to answer caregiver questions
Care coordination	28^44^, ^48^, ^50^, ^51^, ^52^, ^53^, ^57^, ^59^, ^60^, ^65^, ^68^, ^70^, ^72^, ^73^, ^74^, ^76^, ^80^, ^82^, ^83^, ^86^, ^87^, ^88^, ^89^, ^91^, ^93^, ^95^, ^96^, ^100^, ^102^	
Adapting a team‐based approach to care delivery	15^44^, ^53^, ^59^, ^60^, ^65^, ^70^, ^82^, ^83^, ^86^, ^87^, ^88^, ^91^, ^95^, ^96^, ^100^	Medical home or ‘one‐stop‐shop’ clinic Coordination of care delivery responsibilities among a multidisciplinary team
Identification of one or more team members to coordinate care	13^48^, ^50^, ^52^, ^57^, ^59^, ^72^, ^73^, ^74^, ^76^, ^80^, ^88^, ^93^, ^95^, ^100^	Identification of a team member to coordinate care professionals and/or liaise with the child and family Adapt a referral system to different providers
Documenting and sharing care plans and progress reports with other providers	11^44^, ^51^, ^53^, ^65^, ^73^, ^74^, ^80^, ^82^, ^83^, ^89^, ^95^, ^102^	Accessible electronic health records where care plans can be shared Provide caregivers with progress reports to share with other providers
Using technology to enhance coordination	8^50^, ^51^, ^53^, ^60^, ^68^, ^82^, ^89^, ^102^	Telemedicine programmes connecting families to multiple remote providers
Respect for child and family	27^42^, ^43^, ^45^, ^46^, ^51^, ^53^, ^57^, ^58^, ^60^, ^64^, ^66^, ^71^, ^76^, ^77^, ^79^, ^80^, ^84^, ^85^, ^86^, ^87^, ^88^, ^90^, ^94^, ^95^, ^96^, ^97^, ^99^, ^101^	
In‐person assessments of child and family values, preferences, and needs	17^42^, ^43^, ^45^, ^51^, ^53^, ^57^, ^60^, ^66^, ^71^, ^77^, ^79^, ^80^, ^85^, ^86^, ^87^, ^88^, ^95^, ^99^	Home visit before intake to assess needs Discussion at initial contact about families' worldview and understanding of child's condition
Communication tools to promote families' expression of their values and needs	6^46^, ^58^, ^64^, ^66^, ^90^, ^97^	Checklists Question prompt lists Mobile applications and gamified tools
Adjusting activities and communication to age, language, and culture	5^76^, ^84^, ^94^, ^96^, ^101^	Translators and interpreters Use child‐friendly language to explain the disease and its symptoms Adapt educational materials to the culture and language of the family
Follow‐up and continuity of care	24^50^, ^52^, ^53^, ^57^, ^59^, ^60^, ^63^, ^70^, ^72^, ^73^, ^74^, ^78^, ^80^, ^83^, ^86^, ^87^, ^88^, ^89^, ^91^, ^93^, ^95^, ^96^, ^100^, ^102^, ^103^	
Ensuring access to follow‐up care	22^50^, ^52^, ^53^, ^57^, ^60^, ^63^, ^70^, ^72^, ^73^, ^74^, ^78^, ^80^, ^83^, ^86^, ^87^, ^88^, ^89^, ^93^, ^95^, ^96^, ^100^, ^102^, ^103^	Contact the family after discharge and schedule follow‐up appointments Provide discharge instructions or follow‐up care plans and review them periodically Assess and address child and family needs regularly after discharge (e.g., medical refills, financial needs)
Facilitating care transitions between different settings or levels of care	8^52^, ^60^, ^70^, ^86^, ^88^, ^91^, ^93^, ^100^	From intensive or critical care to the regular ward From tertiary or secondary care to primary care From inpatient care in the hospital to outpatient care in the community
Designating a team member to support follow‐up and continuity of care	8^52^, ^57^, ^59^, ^63^, ^73^, ^74^, ^80^, ^83^, ^95^	Accompany families to outpatient clinic visits after discharge Monitor families' attendance of follow‐up appointments
Emotional support	18^47^, ^53^, ^54^, ^57^, ^60^, ^61^, ^63^, ^64^, ^75^, ^79^, ^81^, ^84^, ^86^, ^87^, ^88^, ^91^, ^95^, ^101^	
Designating an individual or group to provide emotional, psychological, or spiritual support	11^47^, ^53^, ^54^, ^57^, ^60^, ^63^, ^75^, ^86^, ^87^, ^88^, ^91^	Individual or group counselling by psychologists, social workers, health care providers or the clergy
Making changes to the visit process or adjusting the environment of the clinical setting	6^53^, ^61^, ^75^, ^81^, ^84^, ^95^	Create child‐friendly spaces using toys and drawings Expedite the admission process to prevent busy and stressful waiting rooms
Physical comfort	7^53^, ^61^, ^64^, ^75^, ^81^, ^84^, ^88^	
Making changes to the visit process or adjusting the environment of the clinical setting	6^53^, ^61^, ^75^, ^81^, ^84^, ^88^	Give families light control or minimizing bright lights Schedule surgeries early in the morning to prevent long fasting times
Addressing or minimizing physical pain	2^64^, ^88^	Tools that allow children to communicate their pain symptoms and location to providers Incorporate pain management strategies (e.g., medications, relaxation training) into the care plan

## 4 DISCUSSION

### 4.1 Summary of findings

This scoping review aimed to identify and characterize recently described interventions designed to make care more family‐centred for children with chronic conditions and their families, so that practice and research can build on a synthesis and an understanding of how interventions have been developed to date, the domains of family‐centred care that these interventions have addressed and the key components of the interventions that have sought to address those domains. This was an important evidence gap, particularly since family‐centred care is widely agreed to be important but is broadly defined in the literature, rendering it challenging to understand which approaches have been developed to address specific domains, paediatric chronic disease patient populations and settings.

We identified 63 articles published over the course of fewer than 20 months that described family‐centred care interventions for children with chronic conditions. They encompassed a broad range of specific aims related to improving aspects of family‐centred care, activities and methods. Confirming family‐centred care as a multifaceted concept, nearly half of the included interventions addressed at least four family‐centred care domains while only a few addressed a single domain. Communication and information provision, access to care and family involvement were the most common domains addressed by included interventions' aims and activities, though all eight of the domains that we considered, based on the Picker principles of patient‐centred care, were addressed by multiple interventions.

Clear and comprehensive reporting of interventions is important^40^; therefore, we assessed the reporting of included interventions using the TIDieR checklist (Appendices S6 and S7). In our assessment, reporting of many details recommended by the TIDieR checklist was strong, with >85% of studies adequately reporting an intervention name; a rationale, theory or goal of the intervention's elements; activities or procedures; facilitators or providers; and setting. We note that reporting of the intervention's goals and activities were among the inclusion criteria for our review, which likely led to a sample of interventions that were relatively better defined. We identified room for improvement in the reporting of intervention materials, mode of delivery, intervention timing and duration, planned tailoring and actual modifications.

Family and patient co‐design of interventions, particularly those aiming to be family‐centred, broadens the range of ideas and perspectives in design and may improve intervention effectiveness.^18^, ^19^ For close to 30% of included interventions, authors did not report or cite information on the groups engaged in the development of interventions. We anticipated a higher proportion as engagement of health care providers or administrators and of families in the *development or research* of an intervention was an inclusion criterion. Among the interventions that described such involvement in development, caregivers and child patients were reported to have been engaged in the development of 72.7% and 34.8% of the interventions, respectively. There is still room for improvement in engaging these groups in the development of family‐centred care interventions and the reporting of such engagement, particularly children, even with consideration of age and developmental capacity. There may also be a paucity of interventions addressing the needs of siblings of children with chronic conditions. Although 10 interventions described ‘families’ as a target of their activities, we identified only one intervention in which activities directly targeted siblings.^47^ Two additional interventions included advising or educating caregivers to be aware of siblings' emotional needs after a diagnosis or medical event (*data not shown*).^56^, ^88^ Healthy siblings of children with chronic conditions may be more likely to experience adverse psychological effects, especially if the condition is more severe.^133^ More interventions that directly address the needs of siblings are an important part of current efforts to make care more family‐centred.

### 4.2 Coordination of care among providers and with the family

Care coordination is a particularly important aspect of family‐centred care for children with chronic conditions, who often have ongoing relationships with care providers, regularly interact with multiple facets of the health care system beyond primary care, and need daily at‐home care management.^134^ Health care systems often perform poorly in meeting the needs of this population, with fragmented or siloed institutions and providers and a lack of mechanisms for coordination of care and appointments and sharing of information across providers and systems.^6^, ^134^, ^135^ Preferred approaches to health care coordination for children with chronic conditions that have been described include team‐based organization of care, designation of a care coordinator, digital means of information sharing among providers, care plans and patient registries to track and monitor patients.^136^, ^137^ Our review indicates that recent interventions adhere to those recommendations, as we identified four common approaches to address care coordination: medical home or team‐based models; designation of individuals or teams to coordinate aspects of care; processes for sharing information among providers; and using technology to enhance coordination. The most common of these was the medical home or team‐based model, which was examined or described by nearly a quarter of the interventions we reviewed. Such models aim to provide comprehensive, coordinated and accessible care, often through primary care,^138^ though interventions we reviewed that addressed care coordination did so in both hospital and community settings. Several interventions identified one or more care team members to conduct care coordination activities or streamline a referral process. When these team members liaise directly with families, such points of contact also provide an opportunity for relational continuity, building familiarity and trust between families and health care teams. This continuity is consistently viewed as important to families of children with ongoing health care needs.^135^, ^139^

### 4.3 Technology for family‐centred care

The use of technology to improve family‐centred care was common among included interventions; for example, to facilitate access to care, communication or care coordination. However, the interventions included were likely developed and/or studied before the declaration of the COVID‐19 outbreak as a global pandemic on 11 March 2020: our searches were conducted shortly thereafter on 11 August 2020 (published literature) or 7–20 October 2020 (grey literature). Further, among articles that reported intervention implementation dates, all such dates occurred before 11 March 2020. Therefore, these interventions may need to be examined with consideration of needs and realities that have emerged as a result of the pandemic. Web‐based health care interactions, information sharing and education are becoming increasingly common; telehealth increased during the pandemic^140^, ^141^, ^142^ and is likely to remain as a permanent facet of health care delivery in many parts of the world. Parent and family satisfaction with virtual health care or digital health interventions varies and may depend on the context of use.^143^, ^144^, ^145^, ^146^ Moreover, families with low digital literacy or without consistent access to high‐speed Internet may not have equal access to, or equally benefit from, technology‐based interventions, thereby increasing potential inequities in receiving family‐centred care.^147^, ^148^

### 4.4 Strengths and limitations

The high number of published articles returned in our search highlighted a need for a comprehensive, good‐quality review about family‐centred care interventions to provide policymakers, clinicians and researchers engaged in health care improvement for children with chronic conditions and their families with a useful summary of the literature. To create this resource of family‐centred care interventions, we aimed to identify and describe the scope of existing family‐centred care interventions, mapping their component activities and processes within established domains. We used rigorous methods, particularly in the search strategy and screening and extraction of articles, for example, including multiple reviewers and extractors to minimize bias; and we relied on widely accepted principles of family‐centred care to synthesize our findings.

We implemented two deviations from our published protocol: (i) we described the planned review as ‘rapid’ in our protocol but at this reporting stage we believe that the 2‐year timeframe needed to complete it renders that description inaccurate; and (ii) we changed the eligibility criteria regarding publication date for feasibility reasons. With respect to (ii) family‐centred care is a broad concept, necessitating a search strategy that was nonspecific and initially yielded a large number of citations (>15,000). Health care systems and paradigms of care change frequently, however, and our choice to narrow the eligibility to publications since 2019 enabled us to identify interventions that address the most current contexts and needs. The exclusion of articles published before 2019, as well as those published in languages other than English, may introduce selection bias into our review.^149^ Interested readers may refer to Appendix S9 for a list of excluded non‐English articles. The English‐language eligibility criterion also likely affected the geographic distribution of articles included in this review: more than two‐thirds of articles described interventions implemented in North America or the United Kingdom. Finally, this review focused on a broad topic, interventions to improve family‐centred care. We strove to identify and include interventions of interest but some relevant interventions may have been missed or described in article types (e.g., commentaries) that were ineligible for our review.

## 5 CONCLUSION

As recognition of the unmet needs of children with chronic conditions grows, so does interest in improving family‐centred care. The identification of 61 family‐centred care interventions for children with chronic conditions, described in articles published 2019 to mid‐2020, demonstrates that this is an active area of research. The breadth of the concept of family‐centred care may present a challenge for individuals and groups interested in developing and evaluating interventions who want to build on previous work. This review is a concrete resource for health care providers, administrators and researchers, providing an inventory of interventions categorized by family‐centred care domain, setting and population, that describes the types of activities and processes that have been developed and/or implemented recently. It serves as a foundation for those engaged in practice and/or research to improve health care for children with high needs and their families by highlighting interventions that centre the needs of children and their caregivers, and to potentially advocate to governments and funding agencies for the resources to improve that care. The many interventions that have been the subject of RCTs and quasiexperimental designs underscores an opportunity for future systematic reviews to evaluate the effectiveness of interventions for a subset of domains, populations or settings, using our inventory as a starting point. Finally, we have highlighted two areas where future research on the development of future family‐centred care interventions may be improved: (1) involving patients and families in the development process and (2) improving the transparent reporting of intervention development and implementation, particularly with respect to clarifying aims and processes of engaging with patients, caregivers and providers.

## AUTHOR CONTRIBUTIONS

Beth K. Potter, Andrea J. Chow, Zobaida Al‐Baldawi, Ryan Iverson, Isabel Jordan, Nicole Pallone, Maureen Smith, Becky Skidmore, Jamie Brehaut, Pranesh Chakraborty, Eyal Cohen, Sarah Dyack, Jane Gillis, Cheryl R. Greenberg, Robin Hayeems, Brian Hutton, Sara Khangura, Jennifer J. MacKenzie, John J. Mitchell, Stuart G. Nicholls, Amy Pender, Chitra Prasad, Andreas Schulze, Rebecca N. Sparkes, Kathy N. Speechley, Sylvia Stockler, Mari Teitlebaum, Yannis Trakadis, Clara Van Karnebeek, Jagdeep S. Walia and Kumanan Wilson contributed to the conception and design of the study. Becky Skidmore conducted the literature search. Andrea J. Chow, Zobaida Al‐Baldawi, Ryan Iverson, Ammar Saad, Beth K. Potter, and Pranesh Chakraborty contributed to the study selection, and Ammar Saad, Andrea J. Chow, Zeinab Moazin, Zobaida Al‐Baldawi and Ryan Iverson contributed to data extraction. Ammar Saad, Andrea J. Chow and Beth K. Potter analysed the data. Andrea J. Chow, Ammar Saad, Beth K. Potter, Zobaida Al‐Baldawi, Eyal Cohen, Sarah Dyack, Jane Gillis, Ryan Iverson, Isabel Jordan, Sharan Goobie, Nicole Pallone, Chitra Prasad, Maureen Smith and Monica Taljaard interpreted the data. Andrea J. Chow, Beth K. Potter and Ammar Saad drafted the manuscript. All authors critically revised the manuscript and approved the final version.

## CONFLICTS OF INTEREST STATEMENT

John J. Mitchell has worked with pharmaceutical companies that market products for the treatment of inborn errors. This relationship did not have any impact on the design or review of this paper. Kumanan Wilson is a co‐founder and Chief Scientific Officer of CANImmunize Inc. He served on the Independent Data Monitoring Committee for Medicago and is a member of the Moderna Global Advisory Core Consultancy Group. The remaining authors declare no conflict of interest.

## Supporting information

Supporting information.

Supporting information.

Supporting information.

Supporting information.

Supporting information.

Supporting information.

Supporting information.

Supporting information.

Supporting information.

## Data Availability

The data that support the findings of this study are available from the corresponding author upon reasonable request.
