# Supplementary material for: Family‐centred care interventions for children with chronic conditions: A scoping review
Source: Health Expect. 2024 Feb 2;27(1):e13897. doi: 10.1111/hex.13897 (PMC10837485; doi:10.1111/hex.13897)
Supplement: Supplementary file 2 — Supporting information. [file HEX-27-e13897-s008.docx]

**Appendix 2. Database search strategies**

Final Strategy

2020 Aug 11

Ovid Multifile

Database: Embase Classic+Embase <1947 to 2020 August 10> , Ovid MEDLINE(R) ALL <1946 to August 10, 2020>, APA PsycInfo <1806 to August Week 1 2020>, EBM Reviews - Cochrane Central Register of Controlled Trials <July 2020>, EBM Reviews - Cochrane Database of Systematic Reviews <2005 to August 07, 2020>, EBM Reviews - Database of Abstracts of Reviews of Effects <1st Quarter 2016>, EBM Reviews - Health Technology Assessment <4th Quarter 2016>, EBM Reviews - NHS Economic Evaluation Database <1st Quarter 2016>

Search Strategy:

--------------------------------------------------------------------------------

1 Disabled Children/ (15462)

2 exp Infant/ (2335029)

3 exp Child/ (4999075)

4 (baby or babies or infant? or infanc* or neonat* or newborn* or preschool* or pre-school* or toddler?).tw,kf. (2020348)

5 (child* or pre-adolescen* or preteen* or pre-teen* or school-age*).tw,kf. (4216938)

6 (pediatric* or paediatric* or infan* or child*).jn,jw. (1659063)

7 exp Pediatrics/ (207848)

8 p?ediatric*.tw,kf. (1004980)

9 or/2-8 [INFANTS/CHILDREN/PAEDIATRICS] (8365752)

10 exp Chronic Disease/ (479753)

11 (chronic* adj3 (condition? or disease? or disorder* or health or syndrome?)).tw,kf. (963299)

12 chronic* ill*.tw,kf. (65509)

13 chronic* sick*.tw,kf. (931)

14 Amputees/ (4788)

15 amputee?.tw,kf. (15071)

16 Disabled Persons/ (76597)

17 Mentally Disabled Persons/ (4617)

18 Mentally ill Persons/ (35440)

19 Persons with Hearing Impairments/ (3872)

20 Visually Impaired Persons/ (11402)

21 (deformit* or disabled or disabilit* or handicap*).tw,kf. (901928)

22 ((mentally or intellectually or physically) adj1 challenged).tw,kf. (712)

23 (complex* adj2 (medical* or care* or health or healthcare or need?)).tw,kf. (47525)

24 (medical* adj2 fragil*).tw,kf. (833)

25 (special adj2 need*).tw,kf. (33139)

26 (technolog* adj2 depend*).tw,kf. (2534)

27 (frequen* adj2 (bowel movement? or diarrhea or ear infection? or severe headache? or migraine?)).tw,kf. (9625)

28 exp Anemia/ (582983)

29 (an?emia* or an?emi* or thalass?emi*).tw,kf. (459337)

30 ((HbS or h?emoglobin s or sickle cell or sickling) adj2 (condition? or disease? or disorder?)).tw,kf. (41978)

31 Arthritis/ (120518)

32 Arthritis, Juvenile/ (24076)

33 (arthriti* or periarthriti* or peri arthriti* or polyarthriti* or poly arthriti*).tw,kf. (508251)

34 exp Asthma/ (422007)

35 asthma*.tw,kf. (442714)

36 exp "Attention Deficit and Disruptive Behavior Disorders"/ (97543)

37 (attention deficit adj3 disorder?).tw,kf. (97955)

38 (ADDH or ADHD).tw,kf. (95549)

39 (hyperkinetic syndrome? or minimal brain dysfunction?).tw,kf. (3000)

40 exp Autism Spectrum Disorder/ (146506)

41 (autis* or asperger* or kanner* syndrome?).tw,kf. (173318)

42 Problem Behavior/ (8073)

43 ((behavio* or conduct) adj2 (problem? or disruptive or dysfunctional)).tw,kf. (124335)

44 Brain Injuries/ (103268)

45 exp Brain Damage, Chronic/ (44515)

46 exp Brain Injury, Chronic/ (193249)

47 (brain* adj3 (injur* or commotio* or concuss* or damag* or lacerat* or trauma*)).tw,kf. (297946)

48 (commotio or concussion* or TBI or TBIs).tw,kf. (102973)

49 exp Bronchitis/ (103387)

50 (bronchit* or bronchiolit*).tw,kf. (90591)

51 Cerebral Palsy/ (68464)

52 (cerebral palsy or (diplegia adj1 spastic) or Little* disease).tw,kf. (69572)

53 ((brain? or central*) adj1 palsy).tw,kf. (253)

54 ((brain or central* or cerebral*) adj1 (paralys* or pares#s)).tw,kf. (1799)

55 Congenital Abnormalities/ (45985)

56 ((congenital* or birth) adj2 (abnormal* or anomal* or defect* or deform* or malform*)).tw,kf. (198363)

57 Cystic Fibrosis/ (113324)

58 cystic fibros*.tw,kf. (120258)

59 ((fibrocystic or fibro-cystic) adj3 pancrea*).tw,kf. (93)

60 mucoviscidos*.tw,kf. (3462)

61 Depression/ (531505)

62 exp Depressive Disorder/ (609237)

63 (depress* or melanchol*).tw,kf. (1517456)

64 Developmental Disabilities/ (45843)

65 (development* adj2 (delay* or deviat* or disabilit* or disabled or disorder?)).tw,kf. (136333)

66 Diabetes Mellitus/ (724738)

67 Diabetes Mellitus, Type 1/ (114768)

68 ((autoimmune or brittle or insulin-dependent or juvenile) adj3 diabet*).tw,kf. (79852)

69 (("Type 1" or "Type I" or ID) adj DM).tw,kf. (3875)

70 (IDDM or T1D).tw,kf. (38172)

71 Down Syndrome/ (68765)

72 (Down* adj2 syndrome*).tw,kf. (61144)

73 (mongolism* or mongoloid*).tw,kf. (7423)

74 ("trisomy 21" or "trisomy G1" or "trisomy (G)1" or "trisomy G-1" or "trisomy GM" or "trisomy G" or "21 trisomy" or "G1 trisomy" or "G(1) trisomy" or "G-1 trisomy" or "GM trisomy" or "G trisomy").tw,kf. (15751)

75 "Chromosomes, Human, Pair 21"/ (10414)

76 (translocat* adj1 DS).tw,kf. (11)

77 Trisomy/ (24048)

78 ((chromosom* adj1 triplicat*) or trisom*).tw,kf. (49725)

79 exp Epilepsy/ (401197)

80 (epileps* or epilept*).tw,kf. (402245)

81 seizure*.tw,kf. (353089)

82 convulsi*.tw,kf. (77536)

83 falling sickness*.tw,kf. (65)

84 comitial disease*.tw,kf. (3)

85 (petit mal or grand mal or absence status).tw,kf. (8472)

86 Dravet* Syndrome*.tw,kf. (3073)

87 Landau-Kleffner* Syndrome*.tw,kf. (1301)

88 Lennox Gastaut* Syndrome*.tw,kf. (4114)

89 Doose* syndrome*.tw,kf. (202)

90 Ohtahara* syndrome*.tw,kf. (601)

91 Sturge-Weber* Syndrome*.tw,kf. (3136)

92 ((sturge* or weber*) adj2 (disease* or syndrome*)).tw,kf. (8732)

93 ((West or "West's") adj syndrome*).tw,kf. (3627)

94 (myoclonic encephalopath* or action myoclonus-renal failure syndrome* or atypical inclusion-body disease* or biotin-responsive encephalopath* or haw river syndrome* or may white syndrome* or myoclonus-nephropathy syndrome* or naito oyanagi disease*).tw,kf. (895)

95 SMEI.tw,kf. (478)

96 (MERRF or fukuhara disease* or fukuhara disorder* or myoencephalopathy ragged-red fiber disease* or myoencephalopathy ragged-red fibre disease*).tw,kf. (1209)

97 Lafora.tw,kf. (1561)

98 ((Unverricht* adj1 Lundborg) or Baltic Myoclonus or Unverricht disease* or Unverricht* syndrome*).tw,kf. (799)

99 ((infantile or nodding) adj2 spasm?).tw,kf. (6966)

100 ((flexor or "in flexion") adj2 spasm?).tw,kf. (367)

101 ((lightning or salaam) adj2 attack?).tw,kf. (17)

102 hypsarrhythmi*.tw,kf. (2358)

103 Heart Diseases/ (108757)

104 exp Heart Defects, Congenital/ (307179)

105 ((cardiac* or cardio* or heart?) adj2 (abnormalit* or anomal* or atypical* or a-typical* or defect* or deficien* or deform* or disorder? or impair* or malform*)).tw,kf. (167036)

106 (tetralog* adj2 fallot*).tw,kf. (24549)

107 ((cardiac* or cardio* or heart?) adj5 (congenital* or inborn* or hereditar* or inherit*)).tw,kf. (137143)

108 Genetic Diseases, Inborn/ (42813)

109 ((genetic or hereditary or inherited or inborn) adj2 (condition? or disease? or disorder?)).tw,kf. (195426)

110 single gene defect?.tw,kf. (1252)

111 Hemophilia A/ (44757)

112 (h?emophili* or (("factor VIII" or "factor 8") adj3 deficien*)).tw,kf. (66730)

113 Hematologic Diseases/ (33026)

114 ((blood or h?ematologic*) adj2 (condition? or disease? or disorder?)).tw,kf. (78942)

115 exp HIV/ (341001)

116 exp HIV Infections/ (673302)

117 (HIV-1 or HIV1 or HIV-I or HIVI or (HIV adj1 type 1) or (HIV adj1 type I)).tw,kf. (186809)

118 (HIV-2 or HIV2 or HIV-II or HIVII or (HIV adj1 type 2) or (HIV adj1 type II)).tw,kf. (11008)

119 (immunodeficiency virus* adj2 human?).tw,kf. (195185)

120 (immuno-deficiency virus* adj2 human?).tw,kf. (608)

121 ((acquired immunodeficiency or acquired immuno-deficiency) adj2 virus*).tw,kf. (2419)

122 AIDS virus*.tw,kf. (2411)

123 HTLV*.tw,kf. (29702)

124 (LAV-1 or LAV1 or LAV I or LAVI or LAV-2 or LAV2 or LAV II or LAVII).tw,kf. (2877)

125 (human T cell lymphotropic adj2 virus*).tw,kf. (5039)

126 (human T lymphotropic adj2 virus*).tw,kf. (5978)

127 lymphadenopathy associated virus*.tw,kf. (599)

128 exp Hypersensitivity/ (1093461)

129 (allerg* or hypersensitiv*).tw,kf. (681380)

130 Intellectual Disability/ (63412)

131 (intellectual* adj2 (deficit* or delay* or deviation* or disabil* or disabled or disorder* or dysfunction* or handicap* or impair* or retard*)).tw,kf. (72035)

132 (brain* adj2 (deficit* or delay* or deviation* or disabil* or disabled or disorder* or dysfunction* or handicap* or impair* or retard*)).tw,kf. (60728)

133 (cognitiv* adj2 (deficit* or delay* or deviation* or disabil* or disabled or disorder* or dysfunction* or handicap* or impair* or retard*)).tw,kf. (354009)

134 (cognition* adj2 (deficit* or delay* or deviation* or disabil* or disabled or disorder* or dysfunction* or handicap* or impair* or retard*)).tw,kf. (16903)

135 (mental* adj2 (deficit* or delay* or deviation* or disabil* or disabled or disorder* or dysfunction* or handicap* or impair* or retard*)).tw,kf. (337388)

136 exp Kidney Diseases/ (1546323)

137 ((kidney? or renal) adj2 (condition? or disease? or disorder?)).tw,kf. (384998)

138 exp Learning Disabilities/ (87705)

139 ((learning or scholastic*) adj2 (atypical* or a-typical* or deficit* or delay* or disabil* or disabled or disorder* or disturb* or dysfunction* or handicap* or impair* or retard*)).tw,kf. (84925)

140 (acalculi* or dyscalculi* or alexia* or alexic* or dyslexia* or dyslexic* or reading disab* or reading disorder* or word blindness or (verbal* adj1 agnosi*)).tw,kf. (33503)

141 exp Language Development Disorders/ (10923)

142 ((language or auditor* or semantic* or speech or speak* or talk* or verbal*) adj2 (atypical* or a-typical* or deficit* or delay* or disabil* or disabled or disorder* or disturb* or dysfunction* or handicap* or impair* or retard*)).tw,kf. (101496)

143 (agraphia? or anomia? or aprosodi* or cluttering? or dysglossia? or dyslalia? or mutism or rhinolalia? or stutter* or stammer*).tw,kf. (31112)

144 Hearing Disorders/ (29420)

145 exp Hearing Loss/ (182120)

146 (hearing adj2 (damag* or degrad* or difficult* or disorder? or distort* or impair* or lose or loses or losting or loss$2 or lost)).tw,kf. (154838)

147 (deaf or deafness* or dysacus* or hypacus* or hypoacus* or hypo-acus* or paracous* or paracus*).tw,kf. (92264)

148 (SSHL or SSNHL or ISSHL or ISSNHL or SISHL or SISNHL).tw,kf. (2069)

149 (SSHLs or SSNHLs or ISSHLs or ISSNHLs or SISHLs or SISNHLs).tw,kf. (2)

150 exp Blindness/ (74107)

151 (amauros#s or blind*).tw,kf. (1141689)

152 Vision, Low/ (5107)

153 ((vision or visual*) adj2 (damag* or degrad* or difficult* or disorder? or impair* or lose or loses or losing or loss$2 or lost or low)).tw,kf. (145955)

154 Meningomyelocele/ (13271)

155 (meningomyelocele or meningo-myelocele or myelocele).tw,kf. (2710)

156 exp Musculoskeletal Diseases/ (3614267)

157 ((musculoskelet* or musculo-skelet* or muscle? or muscular or skelet* or orthop?edic*) adj2 (abnormalit* or deformit* or disorder? or disease?)).tw,kf. (94618)

158 (MSD? or MSKD?).tw,kf. (13947)

159 exp Neoplasms/ (8406233)

160 (neoplas* or cancer* or tumour* or tumor* or carcinoma* or malignan* or metasta* or oncolog*).tw,kf. (8527394)

161 (adenoma? or adenocarcinoma? or adeno-carcinoma? or blastoma? or carcinosarcoma? or carcino-sarcoma? or hepatoblastoma? or hepato-blastoma? or leukemia? or leukaemia? or lymphoma? or melanoma? or mesenchymoma? or mesothelioma? or sarcoma? or thymoma?).tw,kf. (2062321)

162 exp Spinal Dysraphism/ (21584)

163 ((spina? or status) adj (bifida? or dysraphi*)).tw,kf. (21987)

164 (((cleft or open) adj spine?) or rachischis* or schistorrhach*).tw,kf. (433)

165 Child Development Disorders, Pervasive/ (32020)

166 (development* disorder? adj2 pervasive*).tw,kf. (8346)

167 Mental Disorders/ (310497)

168 ((mental* or psychiatric*) adj1 (disorder? or ill* or disease?)).tw,kf. (479871)

169 (behavio?r* adj1 disorder?).tw,kf. (46768)

170 exp Mood Disorders/ (813276)

171 mood disorder?.tw,kf. (63630)

172 Depression/ (531505)

173 depress*.tw,kf. (1513346)

174 exp Schizophrenia/ (400024)

175 (dementia praecox or schizophreni*).tw,kf. (445861)

176 exp Tic Disorders/ (21241)

177 (tic disorder? or facial twitch* or habit spasm* or nervous twitch*).tw,kf. (5437)

178 tourette*.tw,kf. (16957)

179 or/10-178 [CHRONIC CONDITIONS/DISABILITIES] (25218455)

180 9 and 179 [CHILDREN - CHRONIC CONDITIONS/DISABILITIES] (3767825)

181 1 or 180 [CHILDREN - CHRONIC CONDITIONS/DISABILITIES] (3770342)

182 exp Child Health Services/ (121260)

183 Child Day Care Centers/ (17683)

184 ((care or daycare or healthcare or program? or programme? or service?) adj3 (baby or babies or infant? or infanc* or neonat* or newborn* or preschool* or pre-school* or toddler* or child* or pre-adolescen* or preteen* or pre-teen* or school-age* or p?ediatric*)).tw,kf. (386476)

185 ((intervention* or therap* or treatment* or rehab*) adj3 (baby or babies or infant? or infanc* or neonat* or newborn* or preschool* or pre-school* or toddler* or child* or pre-adolescen* or preteen* or pre-teen* or school-age* or p?ediatric*)).tw,kf. (367792)

186 (early intervention* or head start*).tw,kf. (76032)

187 School Health Services/ (34823)

188 School Mental Health Services/ (42)

189 School Nursing/ (10929)

190 (school? adj3 health service?).tw,kf. (4177)

191 school nursing.tw,kf. (2308)

192 or/182-191 [CHILD HEALTH SERVICES/THERAPIES] (894560)

193 Professional-Family Relations/ (97512)

194 ((care provider* or clinician* or doctor* or HCP or HCPs or physician* or professional* or provider* or therapist*) adj3 (family or families or parent*) adj5 relation*).tw,kf. (3561)

195 Patient-Centered Care/ (205330)

196 ((family or families or parent*) adj3 (centre* or center$2 or support*)).tw,kf. (123828)

197 ((patient or patients) adj3 (centre* or center$2 or support*)).tw,kf. (298899)

198 collaborat*.tw,kf. (467631)

199 (partnering or partnership?).tw,kf. (117772)

200 ((care or healthcare or health care or service? or program? or programme? or relational) adj3 coordinat*).tw,kf. (45959)

201 Patient Satisfaction/ (237760)

202 Patient Preference/ (28064)

203 Patient Participation/ (57022)

204 ((participat* or prefer* or satisf*) adj3 (family* or families or parent*)).tw,kf. (64428)

205 ((participat* or prefer* or satisf*) adj3 (baby or babies or infant? or infanc* or neonat* or newborn* or preschool* or pre-school* or toddler* or child* or pre-adolescen* or preteen* or pre-teen* or school-age* or p?ediatric*)).tw,kf. (71892)

206 ((participat* or prefer* or satisf*) adj3 patient*).tw,kf. (361948)

207 Decision Making, Shared/ (7471)

208 (decision* adj3 (engag* or participat* or partner* or share? or sharing or active* involv*)).tw,kf. (45701)

209 or/193-208 [FAMILY-CENTRED] (1875558)

210 192 and 209 [FAMILY-CENTRED CHILD HEALTH SERVICES] (108013)

211 181 and 210 [CHILDREN - CHRONIC CONDITIONS/DISABILITIES - FAMILY-CENTRED CHILD HEALTH SERVICES] (48346)

212 exp Animals/ not Humans/ (18014151)

213 211 not 212 [ANIMAL-ONLY REMOVED] (39406)

214 (comment or editorial or news or newspaper article).pt. (2101086)

215 (letter not (letter and randomized controlled trial)).pt. (2220420)

216 213 not (214 or 215) [OPINION PIECES REMOVED] (39210)

217 limit 216 to yr="2010-current" [Limit not valid in DARE; records were retained] (23486)

218 217 use medall [MEDLINE RECORDS] (7543)

219 handicapped child/ (16037)

220 exp child/ (4999075)

221 (baby or babies or infant? or infanc* or neonat* or newborn* or preschool* or pre-school* or toddler?).tw,kw. (2011673)

222 (child* or pre-adolescen* or preteen* or pre-teen* or school-age*).tw,kw. (4233670)

223 (pediatric* or paediatric* or infan* or child*).jn,jw. (1659063)

224 exp pediatrics/ (207848)

225 p?ediatric*.tw,kw. (1032769)

226 or/220-225 [INFANTS/CHILDREN/PAEDIATRICS] (8223256)

227 exp chronic disease/ (479753)

228 (chronic* adj3 (condition? or disease? or disorder* or health or syndrome?)).tw,kw. (973165)

229 chronic* ill*.tw,kw. (67068)

230 chronic* sick*.tw,kw. (984)

231 exp disabled person/ (127634)

232 amputee?.tw,kw. (15287)

233 (deformit* or disabled or disabilit* or handicap*).tw,kw. (908451)

234 ((mentally or intellectually or physically) adj1 challenged).tw,kw. (710)

235 (complex* adj2 (medical* or care* or health or healthcare or need?)).tw,kw. (47668)

236 (medical* adj2 fragil*).tw,kw. (844)

237 (special adj2 need*).tw,kw. (33270)

238 (technolog* adj2 depend*).tw,kw. (2549)

239 (frequen* adj2 (bowel movement? or diarrhea or ear infection? or severe headache? or migraine?)).tw,kw. (9634)

240 exp anemia/ (582983)

241 (an?emia* or an?emi* or thalass?emi*).tw,kw. (467821)

242 ((HbS or h?emoglobin s or sickle cell or sickling) adj2 (condition? or disease? or disorder?)).tw,kw. (42312)

243 arthritis/ (120518)

244 exp juvenile rheumatoid arthritis/ (33283)

245 (arthriti* or periarthriti* or peri arthriti* or polyarthriti* or poly arthriti*).tw,kw. (514568)

246 exp asthma/ (422007)

247 asthma*.tw,kw. (448846)

248 attention deficit disorder/ (92457)

249 (attention deficit adj3 disorder?).tw,kw. (99454)

250 (ADDH or ADHD).tw,kw. (96584)

251 (hyperkinetic syndrome? or minimal brain dysfunction?).tw,kw. (3044)

252 behavior disorder/ (66735)

253 exp autism/ (136600)

254 (autis* or asperger* or kanner* syndrome?).tw,kw. (175086)

255 exp disruptive behavior/ (43023)

256 ((behavio* or conduct) adj2 (problem? or disruptive or dysfunctional)).tw,kw. (124898)

257 exp brain injury/ (283144)

258 exp chronic brain disease/ (5637)

259 (brain* adj3 (injur* or commotio* or concuss* or damag* or lacerat* or trauma*)).tw,kw. (299616)

260 (commotio or concussion* or TBI or TBIs).tw,kw. (104049)

261 exp bronchitis/ (103387)

262 (bronchit* or bronchiolit*).tw,kw. (92651)

263 cerebral palsy/ (68464)

264 (cerebral palsy or (diplegia adj1 spastic) or Little* disease).tw,kw. (70380)

265 ((brain? or central*) adj1 palsy).tw,kw. (259)

266 ((brain or central* or cerebral*) adj1 (paralys* or pares#s)).tw,kw. (1899)

267 congenital disorder/ (88258)

268 ((congenital* or birth) adj2 (abnormal* or anomal* or defect* or deform* or malform*)).tw,kw. (199543)

269 cystic fibrosis/ (113324)

270 cystic fibros*.tw,kw. (121494)

271 ((fibrocystic or fibro-cystic) adj3 pancrea*).tw,kw. (93)

272 mucoviscidos*.tw,kw. (3534)

273 exp depression/ (644653)

274 (depress* or melanchol*).tw,kw. (1530612)

275 exp developmental disorder/ (46211)

276 (development* adj2 (delay* or deviat* or disabilit* or disabled or disorder?)).tw,kw. (138003)

277 diabetes mellitus/ (724738)

278 exp insulin dependent diabetes mellitus/ (198698)

279 ((autoimmune or brittle or insulin-dependent or juvenile) adj3 diabet*).tw,kw. (95789)

280 (("Type 1" or "Type I" or ID) adj DM).tw,kw. (3905)

281 (IDDM or T1D).tw,kw. (38437)

282 Down syndrome/ (68765)

283 (Down* adj2 syndrome*).tw,kw. (61719)

284 (mongolism* or mongoloid*).tw,kw. (7536)

285 ("trisomy 21" or "trisomy G1" or "trisomy (G)1" or "trisomy G-1" or "trisomy GM" or "trisomy G" or "21 trisomy" or "G1 trisomy" or "G(1) trisomy" or "G-1 trisomy" or "GM trisomy" or "G trisomy").tw,kw. (16317)

286 chromosome 21/ (10414)

287 (translocat* adj1 DS).tw,kw. (11)

288 exp trisomy/ (38945)

289 ((chromosom* adj1 triplicat*) or trisom*).tw,kw. (50582)

290 exp epilepsy/ (401197)

291 (epileps* or epilept*).tw,kw. (408727)

292 seizure*.tw,kw. (356273)

293 convulsi*.tw,kw. (79052)

294 falling sickness*.tw,kw. (68)

295 comitial disease*.tw,kw. (3)

296 (petit mal or grand mal or absence status).tw,kw. (8400)

297 Dravet* Syndrome*.tw,kw. (3130)

298 Landau-Kleffner* Syndrome*.tw,kw. (1345)

299 Lennox Gastaut* Syndrome*.tw,kw. (4202)

300 Doose* syndrome*.tw,kw. (217)

301 Ohtahara* syndrome*.tw,kw. (622)

302 Sturge-Weber* Syndrome*.tw,kw. (3185)

303 ((sturge* or weber*) adj2 (disease* or syndrome*)).tw,kw. (8898)

304 ((West or "West's") adj syndrome*).tw,kw. (3716)

305 (myoclonic encephalopath* or action myoclonus-renal failure syndrome* or atypical inclusion-body disease* or biotin-responsive encephalopath* or haw river syndrome* or may white syndrome* or myoclonus-nephropathy syndrome* or naito oyanagi disease*).tw,kw. (905)

306 SMEI.tw,kw. (502)

307 (MERRF or fukuhara disease* or fukuhara disorder* or myoencephalopathy ragged-red fiber disease* or myoencephalopathy ragged-red fibre disease*).tw,kw. (1245)

308 Lafora.tw,kw. (1574)

309 ((Unverricht* adj1 Lundborg) or Baltic Myoclonus or Unverricht disease* or Unverricht* syndrome*).tw,kw. (803)

310 ((infantile or nodding) adj2 spasm?).tw,kw. (7186)

311 ((flexor or "in flexion") adj2 spasm?).tw,kw. (367)

312 ((lightning or salaam) adj2 attack?).tw,kw. (17)

313 hypsarrhythmi*.tw,kw. (2403)

314 heart disease/ (200387)

315 exp congenital heart malformation/ (153649)

316 ((cardiac* or cardio* or heart?) adj2 (abnormalit* or anomal* or atypical* or a-typical* or defect* or deficien* or deform* or disorder? or impair* or malform*)).tw,kw. (165024)

317 (tetralog* adj2 fallot*).tw,kw. (24414)

318 ((cardiac* or cardio* or heart?) adj5 (congenital* or inborn* or hereditar* or inherit*)).tw,kw. (139346)

319 genetic disorder/ (78177)

320 ((genetic or hereditary or inherited or inborn) adj2 (condition? or disease? or disorder?)).tw,kw. (197082)

321 single gene defect?.tw,kw. (1260)

322 Hemophilia A/ (44757)

323 (h?emophili* or (("factor VIII" or "factor 8") adj3 deficien*)).tw,kw. (67329)

324 hematologic disease/ (46500)

325 ((blood or h?ematologic*) adj2 (condition? or disease? or disorder?)).tw,kw. (80335)

326 exp human immunodeficiency virus/ (341001)

327 exp human immunodeficiency virus infection/ (377147)

328 (HIV-1 or HIV1 or HIV-I or HIVI or (HIV adj1 type 1) or (HIV adj1 type I)).tw,kw. (188220)

329 (HIV-2 or HIV2 or HIV-II or HIVII or (HIV adj1 type 2) or (HIV adj1 type II)).tw,kw. (11075)

330 (immunodeficiency virus* adj2 human?).tw,kw. (204452)

331 (immuno-deficiency virus* adj2 human?).tw,kw. (629)

332 ((acquired immunodeficiency or acquired immuno-deficiency) adj2 virus*).tw,kw. (2453)

333 AIDS virus*.tw,kw. (2424)

334 HTLV*.tw,kw. (29940)

335 (LAV-1 or LAV1 or LAV I or LAVI or LAV-2 or LAV2 or LAV II or LAVII).tw,kw. (2878)

336 (human T cell lymphotropic adj2 virus*).tw,kw. (5077)

337 (human T lymphotropic adj2 virus*).tw,kw. (6065)

338 lymphadenopathy associated virus*.tw,kw. (599)

339 exp hypersensitivity/ (1093461)

340 (allerg* or hypersensitiv*).tw,kw. (691958)

341 intellectual impairment/ (28260)

342 (intellectual* adj2 (deficit* or delay* or deviation* or disabil* or disabled or disorder* or dysfunction* or handicap* or impair* or retard*)).tw,kw. (72593)

343 (brain* adj2 (deficit* or delay* or deviation* or disabil* or disabled or disorder* or dysfunction* or handicap* or impair* or retard*)).tw,kw. (61628)

344 (cognitiv* adj2 (deficit* or delay* or deviation* or disabil* or disabled or disorder* or dysfunction* or handicap* or impair* or retard*)).tw,kw. (356640)

345 (cognition* adj2 (deficit* or delay* or deviation* or disabil* or disabled or disorder* or dysfunction* or handicap* or impair* or retard*)).tw,kw. (17940)

346 (mental* adj2 (deficit* or delay* or deviation* or disabil* or disabled or disorder* or dysfunction* or handicap* or impair* or retard*)).tw,kw. (333891)

347 exp kidney disease/ (1545756)

348 ((kidney? or renal) adj2 (condition? or disease? or disorder?)).tw,kw. (385481)

349 exp learning disorder/ (72090)

350 ((learning or scholastic*) adj2 (atypical* or a-typical* or deficit* or delay* or disabil* or disabled or disorder* or disturb* or dysfunction* or handicap* or impair* or retard*)).tw,kw. (85497)

351 (acalculi* or dyscalculi* or alexia* or alexic* or dyslexia* or dyslexic* or reading disab* or reading disorder* or word blindness or (verbal* adj1 agnosi*)).tw,kw. (34090)

352 exp developmental language disorder/ (10923)

353 ((language or auditor* or semantic* or speech or speak* or talk* or verbal*) adj2 (atypical* or a-typical* or deficit* or delay* or disabil* or disabled or disorder* or disturb* or dysfunction* or handicap* or impair* or retard*)).tw,kw. (101234)

354 (agraphia? or anomia? or aprosodi* or cluttering? or dysglossia? or dyslalia? or mutism or rhinolalia? or stutter* or stammer*).tw,kw. (31235)

355 hearing disorder/ (33877)

356 exp hearing impairment/ (182120)

357 (hearing adj2 (damag* or degrad* or difficult* or disorder? or distort* or impair* or lose or loses or losting or loss$2 or lost)).tw,kw. (155613)

358 (deaf or deafness* or dysacus* or hypacus* or hypoacus* or hypo-acus* or paracous* or paracus*).tw,kw. (94045)

359 (SSHL or SSNHL or ISSHL or ISSNHL or SISHL or SISNHL).tw,kw. (2072)

360 (SSHLs or SSNHLs or ISSHLs or ISSNHLs or SISHLs or SISNHLs).tw,kw. (2)

361 exp blindness/ (74107)

362 (amauros#s or blind*).tw,kw. (1163684)

363 low vision/ (6536)

364 ((vision or visual*) adj2 (damag* or degrad* or difficult* or disorder? or impair* or lose or loses or losing or loss$2 or lost or low)).tw,kw. (157311)

365 meningomyelocele/ (13271)

366 (meningomyelocele or meningo-myelocele or myelocele).tw,kw. (2990)

367 exp musculoskeletal disease/ (3614267)

368 ((musculoskelet* or musculo-skelet* or muscle? or muscular or skelet* or orthop?edic*) adj2 (abnormalit* or deformit* or disorder? or disease?)).tw,kw. (96594)

369 (MSD? or MSKD?).tw,kw. (14051)

370 exp neoplasm/ (8400999)

371 (neoplas* or cancer* or tumour* or tumor* or carcinoma* or malignan* or metasta* or oncolog*).tw,kw. (8561222)

372 (adenoma? or adenocarcinoma? or adeno-carcinoma? or blastoma? or carcinosarcoma? or carcino-sarcoma? or hepatoblastoma? or hepato-blastoma? or leukemia? or leukaemia? or lymphoma? or melanoma? or mesenchymoma? or mesothelioma? or sarcoma? or thymoma?).tw,kw. (2074571)

373 exp spinal dysraphism/ (21584)

374 ((spina? or status) adj (bifida? or dysraphi*)).tw,kw. (22316)

375 (((cleft or open) adj spine?) or rachischis* or schistorrhach*).tw,kw. (442)

376 (development* disorder? adj2 pervasive*).tw,kw. (8721)

377 mental disease/ (247198)

378 ((mental* or psychiatric*) adj1 (disorder? or ill* or disease?)).tw,kw. (479408)

379 (behavio?r* adj1 disorder?).tw,kw. (47951)

380 exp mood disorder/ (812895)

381 mood disorder?.tw,kw. (66329)

382 exp depression/ (644653)

383 depress*.tw,kw. (1526480)

384 exp schizophrenia/ (400024)

385 (dementia praecox or schizophreni*).tw,kw. (452364)

386 exp tic/ (16520)

387 (tic disorder? or facial twitch* or habit spasm* or nervous twitch*).tw,kw. (5543)

388 tourette*.tw,kw. (17229)

389 or/227-388 [CHRONIC CONDITIONS/DISABILITIES] (25127763)

390 226 and 389 [CHILDREN - CHRONIC CONDITIONS/DISABILITIES] (3694052)

391 219 or 390 [CHILDREN - CHRONIC CONDITIONS/DISABILITIES] (3694173)

392 exp child health care/ (96549)

393 day care/ (17527)

394 ((care or daycare or healthcare or program? or programme? or service?) adj3 (baby or babies or infant? or infanc* or neonat* or newborn* or preschool* or pre-school* or toddler* or child* or pre-adolescen* or preteen* or pre-teen* or school-age* or p?ediatric*)).tw,kw. (389218)

395 ((intervention* or therap* or treatment* or rehab*) adj3 (baby or babies or infant? or infanc* or neonat* or newborn* or preschool* or pre-school* or toddler* or child* or pre-adolescen* or preteen* or pre-teen* or school-age* or p?ediatric*)).tw,kw. (375195)

396 (early intervention* or head start*).tw,kw. (77872)

397 school health service/ (35487)

398 school mental health service/ (41)

399 school health nursing/ (5756)

400 (school? adj3 health service?).tw,kw. (4734)

401 school nursing.tw,kw. (2470)

402 or/392-401 [CHILD HEALTH SERVICES/THERAPIES] (890761)

403 human relation/ (95040)

404 ((care provider* or clinician* or doctor* or HCP or HCPs or physician* or professional* or provider* or therapist*) adj3 (family or families or parent*) adj5 relation*).tw,kw. (3867)

405 ((family or families or parent*) adj3 (centre* or center$2 or support*)).tw,kw. (123970)

406 ((patient or patients) adj3 (centre* or center$2 or support*)).tw,kw. (300225)

407 collaborat*.tw,kw. (469248)

408 (partnering or partnership?).tw,kw. (118329)

409 ((care or healthcare or health care or service? or program? or programme? or relational) adj3 coordinat*).tw,kw. (46189)

410 patient satisfaction/ (237760)

411 patient preference/ (28064)

412 patient participation/ (57022)

413 ((participat* or prefer* or satisf*) adj3 (family* or families or parent*)).tw,kw. (64867)

414 ((participat* or prefer* or satisf*) adj3 (baby or babies or infant? or infanc* or neonat* or newborn* or preschool* or pre-school* or toddler* or child* or pre-adolescen* or preteen* or pre-teen* or school-age* or p?ediatric*)).tw,kw. (72447)

415 ((participat* or prefer* or satisf*) adj3 patient*).tw,kw. (367915)

416 shared decision making/ (9658)

417 (decision* adj3 (engag* or participat* or partner* or share? or sharing or active* involv*)).tw,kw. (46104)

418 or/403-417 [FAMILY-CENTRED] (1722567)

419 402 and 418 [FAMILY-CENTRED CHILD HEALTH SERVICES] (102846)

420 391 and 419 [CHILDREN - CHRONIC CONDITIONS/DISABILITIES - FAMILY-CENTRED CHILD HEALTH SERVICES] (46469)

421 exp animal/ or exp animal experimentation/ or exp animal model/ or exp animal experiment/ or nonhuman/ or exp vertebrate/ (54155937)

422 exp human/ or exp human experimentation/ or exp human experiment/ (41792816)

423 421 not 422 (12364887)

424 420 not 423 [ANIMAL-ONLY REMOVED] (46409)

425 editorial.pt. (1200008)

426 letter.pt. not (letter.pt. and randomized controlled trial/) (2220337)

427 424 not (425 or 426) [OPINION PIECES REMOVED] (46279)

428 limit 427 to yr="2010-current" [Limit not valid in DARE; records were retained] (31031)

429 conference abstract.pt. (3860418)

430 428 not 429 [CONFERENCE ABSTRACTS REMOVED] (25101)

431 430 use emczd [EMBASE RECORDS] (8928)

432 exp Childhood Development/ (105914)

433 (baby or babies or infant? or infanc* or neonat* or newborn* or preschool* or pre-school* or toddler?).tw,id. (1970198)

434 (child* or pre-adolescen* or preteen* or pre-teen* or school-age*).tw,id. (4169583)

435 (pediatric* or paediatric* or infan* or child*).jn,jw. (1659063)

436 exp Pediatrics/ (207848)

437 p?ediatric*.tw,id. (988967)

438 or/432-437 [INFANTS/CHILDREN/PAEDIATRICS] (6446747)

439 Chronic Illness/ (488074)

440 Chronically Ill Children/ (364)

441 "Chronicity (Disorders)"/ (4448)

442 (chronic* adj3 (condition? or disease? or disorder* or health or syndrome?)).tw,id. (955546)

443 chronic* ill*.tw,id. (64716)

444 chronic* sick*.tw,id. (930)

445 amputee?.tw,id. (14787)

446 Disabilities/ (20622)

447 exp Multiple Disabilities/ (1910)

448 Mental Disorders/ (310497)

449 (deformit* or disabled or disabilit* or handicap*).tw,id. (895526)

450 ((mentally or intellectually or physically) adj1 challenged).tw,id. (710)

451 (complex* adj2 (medical* or care* or health or healthcare or need?)).tw,id. (47422)

452 (medical* adj2 fragil*).tw,id. (827)

453 (special adj2 need*).tw,id. (33043)

454 (technolog* adj2 depend*).tw,id. (2518)

455 (frequen* adj2 (bowel movement? or diarrhea or ear infection? or severe headache? or migraine?)).tw,id. (9625)

456 Anemia/ (261256)

457 (an?emia* or an?emi* or thalass?emi*).tw,id. (451780)

458 ((HbS or h?emoglobin s or sickle cell or sickling) adj2 (condition? or disease? or disorder?)).tw,id. (41745)

459 exp Arthritis/ (794416)

460 (arthriti* or periarthriti* or peri arthriti* or polyarthriti* or poly arthriti*).tw,id. (501453)

461 Asthma/ (390348)

462 asthma*.tw,id. (439783)

463 exp Attention Deficit Disorder/ (120438)

464 (attention deficit adj3 disorder?).tw,id. (97461)

465 (ADDH or ADHD).tw,id. (95016)

466 (hyperkinetic syndrome? or minimal brain dysfunction?).tw,id. (2997)

467 exp Autism Spectrum Disorders/ (75368)

468 (autis* or asperger* or kanner* syndrome?).tw,id. (172263)

469 exp Behavior Disorders/ (1395587)

470 Behavior Problems/ (29216)

471 exp Disruptive Behavior Disorders/ (5867)

472 ((behavio* or conduct) adj2 (problem? or disruptive or dysfunctional)).tw,id. (123994)

473 Brain Damage/ (57616)

474 exp Brain Injuries/ (283522)

475 Cognitive Impairment/ (221807)

476 (brain* adj3 (injur* or commotio* or concuss* or damag* or lacerat* or trauma*)).tw,id. (292800)

477 (commotio or concussion* or TBI or TBIs).tw,id. (102011)

478 Bronchial Disorders/ (162)

479 (bronchit* or bronchiolit*).tw,id. (88999)

480 Cerebral Palsy/ (68464)

481 (cerebral palsy or (diplegia adj1 spastic) or Little* disease).tw,id. (68557)

482 ((brain? or central*) adj1 palsy).tw,id. (253)

483 ((brain or central* or cerebral*) adj1 (paralys* or pares#s)).tw,id. (1619)

484 exp Congenital Disorders/ (2778340)

485 ((congenital* or birth) adj2 (abnormal* or anomal* or defect* or deform* or malform*)).tw,id. (190298)

486 Cystic Fibrosis/ (113324)

487 cystic fibros*.tw,id. (119322)

488 ((fibrocystic or fibro-cystic) adj3 pancrea*).tw,id. (89)

489 mucoviscidos*.tw,id. (3402)

490 Anaclitic Depression/ (70)

491 "Depression (Emotion)"/ (156836)

492 exp Major Depression/ (197006)

493 (depress* or melanchol*).tw,id. (1511422)

494 exp Developmental Disabilities/ (81313)

495 (development* adj2 (delay* or deviat* or disabilit* or disabled or disorder?)).tw,id. (135374)

496 Diabetes Mellitus/ (724738)

497 ((autoimmune or brittle or insulin-dependent or juvenile) adj3 diabet*).tw,id. (79460)

498 (("Type 1" or "Type I" or ID) adj DM).tw,id. (3864)

499 (IDDM or T1D).tw,id. (38094)

500 Down's Syndrome/ (68765)

501 (Down* adj2 syndrome*).tw,id. (60913)

502 (mongolism* or mongoloid*).tw,id. (6721)

503 ("trisomy 21" or "trisomy G1" or "trisomy (G)1" or "trisomy G-1" or "trisomy GM" or "trisomy G" or "21 trisomy" or "G1 trisomy" or "G(1) trisomy" or "G-1 trisomy" or "GM trisomy" or "G trisomy").tw,id. (15546)

504 (translocat* adj1 DS).tw,id. (11)

505 Trisomy/ (24048)

506 ((chromosom* adj1 triplicat*) or trisom*).tw,id. (49341)

507 exp Epilepsy/ (401197)

508 (epileps* or epilept*).tw,id. (398041)

509 seizure*.tw,id. (351886)

510 convulsi*.tw,id. (75883)

511 falling sickness*.tw,id. (63)

512 comitial disease*.tw,id. (3)

513 (petit mal or grand mal or absence status).tw,id. (8293)

514 Dravet* Syndrome*.tw,id. (3043)

515 Landau-Kleffner* Syndrome*.tw,id. (1292)

516 Lennox Gastaut* Syndrome*.tw,id. (4088)

517 Doose* syndrome*.tw,id. (192)

518 Ohtahara* syndrome*.tw,id. (590)

519 Sturge-Weber* Syndrome*.tw,id. (3121)

520 ((sturge* or weber*) adj2 (disease* or syndrome*)).tw,id. (8665)

521 ((West or "West's") adj syndrome*).tw,id. (3554)

522 (myoclonic encephalopath* or action myoclonus-renal failure syndrome* or atypical inclusion-body disease* or biotin-responsive encephalopath* or haw river syndrome* or may white syndrome* or myoclonus-nephropathy syndrome* or naito oyanagi disease*).tw,id. (891)

523 SMEI.tw,id. (471)

524 (MERRF or fukuhara disease* or fukuhara disorder* or myoencephalopathy ragged-red fiber disease* or myoencephalopathy ragged-red fibre disease*).tw,id. (1193)

525 Lafora.tw,id. (1546)

526 ((Unverricht* adj1 Lundborg) or Baltic Myoclonus or Unverricht disease* or Unverricht* syndrome*).tw,id. (793)

527 ((infantile or nodding) adj2 spasm?).tw,id. (6883)

528 ((flexor or "in flexion") adj2 spasm?).tw,id. (366)

529 ((lightning or salaam) adj2 attack?).tw,id. (16)

530 hypsarrhythmi*.tw,id. (2315)

531 Heart Diseases/ (108757)

532 ((cardiac* or cardio* or heart?) adj2 (abnormalit* or anomal* or atypical* or a-typical* or defect* or deficien* or deform* or disorder? or impair* or malform*)).tw,id. (158752)

533 (tetralog* adj2 fallot*).tw,id. (24048)

534 ((cardiac* or cardio* or heart?) adj5 (congenital* or inborn* or hereditar* or inherit*)).tw,id. (129806)

535 ((genetic or hereditary or inherited or inborn) adj2 (condition? or disease? or disorder?)).tw,id. (194187)

536 single gene defect?.tw,id. (1251)

537 Hemophilia/ (42543)

538 (h?emophili* or (("factor VIII" or "factor 8") adj3 deficien*)).tw,id. (66087)

539 "Blood and Lymphatic Disorders"/ (1193)

540 ((blood or h?ematologic*) adj2 (condition? or disease? or disorder?)).tw,id. (74477)

541 Sickle Cell Disease/ (60054)

542 exp HIV/ (341001)

543 (HIV-1 or HIV1 or HIV-I or HIVI or (HIV adj1 type 1) or (HIV adj1 type I)).tw,id. (186404)

544 (HIV-2 or HIV2 or HIV-II or HIVII or (HIV adj1 type 2) or (HIV adj1 type II)).tw,id. (10993)

545 (immunodeficiency virus* adj2 human?).tw,id. (193405)

546 (immuno-deficiency virus* adj2 human?).tw,id. (602)

547 ((acquired immunodeficiency or acquired immuno-deficiency) adj2 virus*).tw,id. (2372)

548 AIDS virus*.tw,id. (2409)

549 HTLV*.tw,id. (29630)

550 (LAV-1 or LAV1 or LAV I or LAVI or LAV-2 or LAV2 or LAV II or LAVII).tw,id. (2870)

551 (human T cell lymphotropic adj2 virus*).tw,id. (5026)

552 (human T lymphotropic adj2 virus*).tw,id. (5941)

553 lymphadenopathy associated virus*.tw,id. (599)

554 Allergic Disorders/ (649)

555 (allerg* or hypersensitiv*).tw,id. (673455)

556 exp Intellectual Development Disorder/ (140682)

557 (intellectual* adj2 (deficit* or delay* or deviation* or disabil* or disabled or disorder* or dysfunction* or handicap* or impair* or retard*)).tw,id. (71277)

558 (brain* adj2 (deficit* or delay* or deviation* or disabil* or disabled or disorder* or dysfunction* or handicap* or impair* or retard*)).tw,id. (60569)

559 (cognitiv* adj2 (deficit* or delay* or deviation* or disabil* or disabled or disorder* or dysfunction* or handicap* or impair* or retard*)).tw,id. (351832)

560 (cognition* adj2 (deficit* or delay* or deviation* or disabil* or disabled or disorder* or dysfunction* or handicap* or impair* or retard*)).tw,id. (16658)

561 (mental* adj2 (deficit* or delay* or deviation* or disabil* or disabled or disorder* or dysfunction* or handicap* or impair* or retard*)).tw,id. (327128)

562 exp Kidney Diseases/ (1546323)

563 ((kidney? or renal) adj2 (condition? or disease? or disorder?)).tw,id. (375151)

564 exp Learning Disabilities/ (87705)

565 ((learning or scholastic*) adj2 (atypical* or a-typical* or deficit* or delay* or disabil* or disabled or disorder* or disturb* or dysfunction* or handicap* or impair* or retard*)).tw,id. (84559)

566 (acalculi* or dyscalculi* or alexia* or alexic* or dyslexia* or dyslexic* or reading disab* or reading disorder* or word blindness or (verbal* adj1 agnosi*)).tw,id. (33244)

567 exp Language Disorders/ (155747)

568 ((language or auditor* or semantic* or speech or speak* or talk* or verbal*) adj2 (atypical* or a-typical* or deficit* or delay* or disabil* or disabled or disorder* or disturb* or dysfunction* or handicap* or impair* or retard*)).tw,id. (99782)

569 (agraphia? or anomia? or aprosodi* or cluttering? or dysglossia? or dyslalia? or mutism or rhinolalia? or stutter* or stammer*).tw,id. (30762)

570 Hearing Disorders/ (29420)

571 exp Hearing Loss/ (182120)

572 Cochlear Implants/ (27031)

573 (hearing adj2 (damag* or degrad* or difficult* or disorder? or distort* or impair* or lose or loses or losting or loss$2 or lost)).tw,id. (151973)

574 (deaf or deafness* or dysacus* or hypacus* or hypoacus* or hypo-acus* or paracous* or paracus*).tw,id. (90354)

575 (SSHL or SSNHL or ISSHL or ISSNHL or SISHL or SISNHL).tw,id. (2067)

576 (SSHLs or SSNHLs or ISSHLs or ISSNHLs or SISHLs or SISNHLs).tw,id. (2)

577 Vision Disorders/ (46788)

578 exp Blind/ (54802)

579 (amauros#s or blind*).tw,id. (1140038)

580 Partially Sighted/ (204)

581 ((vision or visual*) adj2 (damag* or degrad* or difficult* or disorder? or impair* or lose or loses or losing or loss$2 or lost or low)).tw,id. (145525)

582 (meningomyelocele or meningo-myelocele or myelocele).tw,id. (2653)

583 exp Musculoskeletal Diseases/ (3614267)

584 ((musculoskelet* or musculo-skelet* or muscle? or muscular or skelet* or orthop?edic*) adj2 (abnormalit* or deformit* or disorder? or disease?)).tw,id. (91619)

585 (MSD? or MSKD?).tw,id. (13860)

586 exp Neoplasms/ (8406233)

587 (neoplas* or cancer* or tumour* or tumor* or carcinoma* or malignan* or metasta* or oncolog*).tw,id. (8452095)

588 (adenoma? or adenocarcinoma? or adeno-carcinoma? or blastoma? or carcinosarcoma? or carcino-sarcoma? or hepatoblastoma? or hepato-blastoma? or leukemia? or leukaemia? or lymphoma? or melanoma? or mesenchymoma? or mesothelioma? or sarcoma? or thymoma?).tw,id. (2042769)

589 Neurodevelopmental Disorders/ (201026)

590 Spina Bifida/ (16816)

591 ((spina? or status) adj (bifida? or dysraphi*)).tw,id. (21380)

592 (((cleft or open) adj spine?) or rachischis* or schistorrhach*).tw,id. (428)

593 Child Development Disorders, Pervasive/ (32020)

594 (development* disorder? adj2 pervasive*).tw,id. (8268)

595 Mental Disorders/ (310497)

596 ((mental* or psychiatric*) adj1 (disorder? or ill* or disease?)).tw,id. (470687)

597 (behavio?r* adj1 disorder?).tw,id. (45797)

598 exp Mood Disorders/ (813276)

599 mood disorder?.tw,id. (62498)

600 depress*.tw,id. (1507237)

601 exp Schizophrenia/ (400024)

602 (dementia praecox or schizophreni*).tw,id. (441126)

603 exp Tic Disorders/ (21241)

604 (tic disorder? or facial twitch* or habit spasm* or nervous twitch*).tw,id. (5388)

605 tourette*.tw,id. (16832)

606 or/439-605 [CHRONIC CONDITIONS/DISABILITIES] (26099934)

607 438 and 606 [CHILDREN - CHRONIC CONDITIONS/DISABILITIES] (3132174)

608 Child Day Care/ (20673)

609 ((care or daycare or healthcare or program? or programme? or service?) adj3 (baby or babies or infant? or infanc* or neonat* or newborn* or preschool* or pre-school* or toddler* or child* or pre-adolescen* or preteen* or pre-teen* or school-age* or p?ediatric*)).tw,id. (383266)

610 ((intervention* or therap* or treatment* or rehab*) adj3 (baby or babies or infant? or infanc* or neonat* or newborn* or preschool* or pre-school* or toddler* or child* or pre-adolescen* or preteen* or pre-teen* or school-age* or p?ediatric*)).tw,id. (366334)

611 Early Intervention/ (36184)

612 (early intervention* or head start*).tw,id. (75364)

613 School Based Intervention/ (18656)

614 (school? adj3 health service?).tw,id. (4071)

615 school nursing.tw,id. (2151)

616 or/608-615 [CHILD HEALTH SERVICES/THERAPIES] (814909)

617 ((care provider* or clinician* or doctor* or HCP or HCPs or physician* or professional* or provider* or therapist*) adj3 (family or families or parent*) adj5 relation*).tw,id. (3505)

618 Client Centered Therapy/ (3968)

619 ((family or families or parent*) adj3 (centre* or center$2 or support*)).tw,id. (123144)

620 ((patient or patients) adj3 (centre* or center$2 or support*)).tw,id. (296867)

621 Collaboration/ (11824)

622 collaborat*.tw,id. (466327)

623 (partnering or partnership?).tw,id. (117235)

624 ((care or healthcare or health care or service? or program? or programme? or relational) adj3 coordinat*).tw,id. (45690)

625 Client Satisfaction/ (5576)

626 Preferences/ (17409)

627 Client Participation/ (2272)

628 ((participat* or prefer* or satisf*) adj3 (family* or families or parent*)).tw,id. (64389)

629 ((participat* or prefer* or satisf*) adj3 (baby or babies or infant? or infanc* or neonat* or newborn* or preschool* or pre-school* or toddler* or child* or pre-adolescen* or preteen* or pre-teen* or school-age* or p?ediatric*)).tw,id. (71866)

630 ((participat* or prefer* or satisf*) adj3 patient*).tw,id. (360013)

631 Group Decision Making/ (3192)

632 (decision* adj3 (engag* or participat* or partner* or share? or sharing or active* involv*)).tw,id. (45065)

633 or/617-632 [FAMILY-CENTRED] (1470122)

634 616 and 633 [FAMILY-CENTRED CHILD HEALTH SERVICES] (91585)

635 607 and 634 [CHILDREN - CHRONIC CONDITIONS/DISABILITIES - FAMILY-CENTRED CHILD HEALTH SERVICES] (43374)

636 limit 635 to yr="2010-current" [Limit not valid in DARE; records were retained] (29284)

637 636 use medall,emczd,coch,cctr,dare,cleed,clhta (24590)

638 636 not 637 [PSYCINFO RECORDS] (4694)

639 Disabled Children/ (15462)

640 exp Infant/ (2335029)

641 exp Child/ (4999075)

642 (baby or babies or infant? or infanc* or neonat* or newborn* or preschool* or pre-school* or toddler?).ti,ab,kw. (2000864)

643 (child* or pre-adolescen* or preteen* or pre-teen* or school-age*).ti,ab,kw. (4203541)

644 (pediatric* or paediatric* or infan* or child*).jn,jw. (1659063)

645 exp Pediatrics/ (207848)

646 p?ediatric*.ti,ab,kw. (1026058)

647 or/640-646 [INFANTS/CHILDREN/PAEDIATRICS] (8353855)

648 exp Chronic Disease/ (479753)

649 (chronic* adj3 (condition? or disease? or disorder* or health or syndrome?)).ti,ab,kw. (967729)

650 chronic* ill*.ti,ab,kw. (65265)

651 chronic* sick*.ti,ab,kw. (978)

652 Amputees/ (4788)

653 amputee?.ti,ab,kw. (15188)

654 Disabled Persons/ (76597)

655 Mentally Disabled Persons/ (4617)

656 Mentally ill Persons/ (35440)

657 Persons with Hearing Impairments/ (3872)

658 Visually Impaired Persons/ (11402)

659 (deformit* or disabled or disabilit* or handicap*).ti,ab,kw. (896104)

660 ((mentally or intellectually or physically) adj1 challenged).ti,ab,kw. (695)

661 (complex* adj2 (medical* or care* or health or healthcare or need?)).ti,ab,kw. (47353)

662 (medical* adj2 fragil*).ti,ab,kw. (831)

663 (special adj2 need*).ti,ab,kw. (32316)

664 (technolog* adj2 depend*).ti,ab,kw. (2516)

665 (frequen* adj2 (bowel movement? or diarrhea or ear infection? or severe headache? or migraine?)).ti,ab,kw. (9555)

666 exp Anemia/ (582983)

667 (an?emia* or an?emi* or thalass?emi*).ti,ab,kw. (466599)

668 ((HbS or h?emoglobin s or sickle cell or sickling) adj2 (condition? or disease? or disorder?)).ti,ab,kw. (42189)

669 Arthritis/ (120518)

670 Arthritis, Juvenile/ (24076)

671 (arthriti* or periarthriti* or peri arthriti* or polyarthriti* or poly arthriti*).ti,ab,kw. (513274)

672 exp Asthma/ (422007)

673 asthma*.ti,ab,kw. (447762)

674 exp "Attention Deficit and Disruptive Behavior Disorders"/ (97543)

675 (attention deficit adj3 disorder?).ti,ab,kw. (96473)

676 (ADDH or ADHD).ti,ab,kw. (95482)

677 (hyperkinetic syndrome? or minimal brain dysfunction?).ti,ab,kw. (3008)

678 exp Autism Spectrum Disorder/ (146506)

679 (autis* or asperger* or kanner* syndrome?).ti,ab,kw. (172998)

680 Problem Behavior/ (8073)

681 ((behavio* or conduct) adj2 (problem? or disruptive or dysfunctional)).ti,ab,kw. (116176)

682 Brain Injuries/ (103268)

683 exp Brain Damage, Chronic/ (44515)

684 exp Brain Injury, Chronic/ (193249)

685 (brain* adj3 (injur* or commotio* or concuss* or damag* or lacerat* or trauma*)).ti,ab,kw. (296164)

686 (commotio or concussion* or TBI or TBIs).ti,ab,kw. (103548)

687 exp Bronchitis/ (103387)

688 (bronchit* or bronchiolit*).ti,ab,kw. (92176)

689 Cerebral Palsy/ (68464)

690 (cerebral palsy or (diplegia adj1 spastic) or Little* disease).ti,ab,kw. (69272)

691 ((brain? or central*) adj1 palsy).ti,ab,kw. (254)

692 ((brain or central* or cerebral*) adj1 (paralys* or pares#s)).ti,ab,kw. (1882)

693 Congenital Abnormalities/ (45985)

694 ((congenital* or birth) adj2 (abnormal* or anomal* or defect* or deform* or malform*)).ti,ab,kw. (198574)

695 Cystic Fibrosis/ (113324)

696 cystic fibros*.ti,ab,kw. (121171)

697 ((fibrocystic or fibro-cystic) adj3 pancrea*).ti,ab,kw. (92)

698 mucoviscidos*.ti,ab,kw. (3530)

699 Depression/ (531505)

700 exp Depressive Disorder/ (609237)

701 (depress* or melanchol*).ti,ab,kw. (1518910)

702 Developmental Disabilities/ (45843)

703 (development* adj2 (delay* or deviat* or disabilit* or disabled or disorder?)).ti,ab,kw. (134739)

704 Diabetes Mellitus/ (724738)

705 Diabetes Mellitus, Type 1/ (114768)

706 ((autoimmune or brittle or insulin-dependent or juvenile) adj3 diabet*).ti,ab,kw. (95520)

707 (("Type 1" or "Type I" or ID) adj DM).ti,ab,kw. (3891)

708 (IDDM or T1D).ti,ab,kw. (38404)

709 Down Syndrome/ (68765)

710 (Down* adj2 syndrome*).ti,ab,kw. (61299)

711 (mongolism* or mongoloid*).ti,ab,kw. (7475)

712 ("trisomy 21" or "trisomy G1" or "trisomy (G)1" or "trisomy G-1" or "trisomy GM" or "trisomy G" or "21 trisomy" or "G1 trisomy" or "G(1) trisomy" or "G-1 trisomy" or "GM trisomy" or "G trisomy").ti,ab,kw. (16211)

713 "Chromosomes, Human, Pair 21"/ (10414)

714 (translocat* adj1 DS).ti,ab,kw. (11)

715 Trisomy/ (24048)

716 ((chromosom* adj1 triplicat*) or trisom*).ti,ab,kw. (50455)

717 exp Epilepsy/ (401197)

718 (epileps* or epilept*).ti,ab,kw. (406855)

719 seizure*.ti,ab,kw. (354575)

720 convulsi*.ti,ab,kw. (78386)

721 falling sickness*.ti,ab,kw. (66)

722 comitial disease*.ti,ab,kw. (3)

723 (petit mal or grand mal or absence status).ti,ab,kw. (8351)

724 Dravet* Syndrome*.ti,ab,kw. (3114)

725 Landau-Kleffner* Syndrome*.ti,ab,kw. (1325)

726 Lennox Gastaut* Syndrome*.ti,ab,kw. (4174)

727 Doose* syndrome*.ti,ab,kw. (210)

728 Ohtahara* syndrome*.ti,ab,kw. (618)

729 Sturge-Weber* Syndrome*.ti,ab,kw. (3180)

730 ((sturge* or weber*) adj2 (disease* or syndrome*)).ti,ab,kw. (8885)

731 ((West or "West's") adj syndrome*).ti,ab,kw. (3687)

732 (myoclonic encephalopath* or action myoclonus-renal failure syndrome* or atypical inclusion-body disease* or biotin-responsive encephalopath* or haw river syndrome* or may white syndrome* or myoclonus-nephropathy syndrome* or naito oyanagi disease*).ti,ab,kw. (883)

733 SMEI.ti,ab,kw. (499)

734 (MERRF or fukuhara disease* or fukuhara disorder* or myoencephalopathy ragged-red fiber disease* or myoencephalopathy ragged-red fibre disease*).ti,ab,kw. (1240)

735 Lafora.ti,ab,kw. (1551)

736 ((Unverricht* adj1 Lundborg) or Baltic Myoclonus or Unverricht disease* or Unverricht* syndrome*).ti,ab,kw. (782)

737 ((infantile or nodding) adj2 spasm?).ti,ab,kw. (7121)

738 ((flexor or "in flexion") adj2 spasm?).ti,ab,kw. (365)

739 ((lightning or salaam) adj2 attack?).ti,ab,kw. (17)

740 hypsarrhythmi*.ti,ab,kw. (2397)

741 Heart Diseases/ (108757)

742 exp Heart Defects, Congenital/ (307179)

743 ((cardiac* or cardio* or heart?) adj2 (abnormalit* or anomal* or atypical* or a-typical* or defect* or deficien* or deform* or disorder? or impair* or malform*)).ti,ab,kw. (163998)

744 (tetralog* adj2 fallot*).ti,ab,kw. (24392)

745 ((cardiac* or cardio* or heart?) adj5 (congenital* or inborn* or hereditar* or inherit*)).ti,ab,kw. (139057)

746 Genetic Diseases, Inborn/ (42813)

747 ((genetic or hereditary or inherited or inborn) adj2 (condition? or disease? or disorder?)).ti,ab,kw. (196402)

748 single gene defect?.ti,ab,kw. (1259)

749 Hemophilia A/ (44757)

750 (h?emophili* or (("factor VIII" or "factor 8") adj3 deficien*)).ti,ab,kw. (67227)

751 Hematologic Diseases/ (33026)

752 ((blood or h?ematologic*) adj2 (condition? or disease? or disorder?)).ti,ab,kw. (79932)

753 exp HIV/ (341001)

754 exp HIV Infections/ (673302)

755 (HIV-1 or HIV1 or HIV-I or HIVI or (HIV adj1 type 1) or (HIV adj1 type I)).ti,ab,kw. (187992)

756 (HIV-2 or HIV2 or HIV-II or HIVII or (HIV adj1 type 2) or (HIV adj1 type II)).ti,ab,kw. (11031)

757 (immunodeficiency virus* adj2 human?).ti,ab,kw. (203212)

758 (immuno-deficiency virus* adj2 human?).ti,ab,kw. (609)

759 ((acquired immunodeficiency or acquired immuno-deficiency) adj2 virus*).ti,ab,kw. (2415)

760 AIDS virus*.ti,ab,kw. (2418)

761 HTLV*.ti,ab,kw. (29923)

762 (LAV-1 or LAV1 or LAV I or LAVI or LAV-2 or LAV2 or LAV II or LAVII).ti,ab,kw. (2864)

763 (human T cell lymphotropic adj2 virus*).ti,ab,kw. (5062)

764 (human T lymphotropic adj2 virus*).ti,ab,kw. (6053)

765 lymphadenopathy associated virus*.ti,ab,kw. (597)

766 exp Hypersensitivity/ (1093461)

767 (allerg* or hypersensitiv*).ti,ab,kw. (684156)

768 Intellectual Disability/ (63412)

769 (intellectual* adj2 (deficit* or delay* or deviation* or disabil* or disabled or disorder* or dysfunction* or handicap* or impair* or retard*)).ti,ab,kw. (70644)

770 (brain* adj2 (deficit* or delay* or deviation* or disabil* or disabled or disorder* or dysfunction* or handicap* or impair* or retard*)).ti,ab,kw. (60628)

771 (cognitiv* adj2 (deficit* or delay* or deviation* or disabil* or disabled or disorder* or dysfunction* or handicap* or impair* or retard*)).ti,ab,kw. (352255)

772 (cognition* adj2 (deficit* or delay* or deviation* or disabil* or disabled or disorder* or dysfunction* or handicap* or impair* or retard*)).ti,ab,kw. (17585)

773 (mental* adj2 (deficit* or delay* or deviation* or disabil* or disabled or disorder* or dysfunction* or handicap* or impair* or retard*)).ti,ab,kw. (303524)

774 exp Kidney Diseases/ (1546323)

775 ((kidney? or renal) adj2 (condition? or disease? or disorder?)).ti,ab,kw. (384431)

776 exp Learning Disabilities/ (87705)

777 ((learning or scholastic*) adj2 (atypical* or a-typical* or deficit* or delay* or disabil* or disabled or disorder* or disturb* or dysfunction* or handicap* or impair* or retard*)).ti,ab,kw. (82226)

778 (acalculi* or dyscalculi* or alexia* or alexic* or dyslexia* or dyslexic* or reading disab* or reading disorder* or word blindness or (verbal* adj1 agnosi*)).ti,ab,kw. (32915)

779 exp Language Development Disorders/ (10923)

780 ((language or auditor* or semantic* or speech or speak* or talk* or verbal*) adj2 (atypical* or a-typical* or deficit* or delay* or disabil* or disabled or disorder* or disturb* or dysfunction* or handicap* or impair* or retard*)).ti,ab,kw. (97786)

781 (agraphia? or anomia? or aprosodi* or cluttering? or dysglossia? or dyslalia? or mutism or rhinolalia? or stutter* or stammer*).ti,ab,kw. (30821)

782 Hearing Disorders/ (29420)

783 exp Hearing Loss/ (182120)

784 (hearing adj2 (damag* or degrad* or difficult* or disorder? or distort* or impair* or lose or loses or losting or loss$2 or lost)).ti,ab,kw. (154163)

785 (deaf or deafness* or dysacus* or hypacus* or hypoacus* or hypo-acus* or paracous* or paracus*).ti,ab,kw. (92826)

786 (SSHL or SSNHL or ISSHL or ISSNHL or SISHL or SISNHL).ti,ab,kw. (2069)

787 (SSHLs or SSNHLs or ISSHLs or ISSNHLs or SISHLs or SISNHLs).ti,ab,kw. (2)

788 exp Blindness/ (74107)

789 (amauros#s or blind*).ti,ab,kw. (1145784)

790 Vision, Low/ (5107)

791 ((vision or visual*) adj2 (damag* or degrad* or difficult* or disorder? or impair* or lose or loses or losing or loss$2 or lost or low)).ti,ab,kw. (155973)

792 Meningomyelocele/ (13271)

793 (meningomyelocele or meningo-myelocele or myelocele).ti,ab,kw. (2978)

794 exp Musculoskeletal Diseases/ (3614267)

795 ((musculoskelet* or musculo-skelet* or muscle? or muscular or skelet* or orthop?edic*) adj2 (abnormalit* or deformit* or disorder? or disease?)).ti,ab,kw. (95932)

796 (MSD? or MSKD?).ti,ab,kw. (13445)

797 exp Neoplasms/ (8406233)

798 (neoplas* or cancer* or tumour* or tumor* or carcinoma* or malignan* or metasta* or oncolog*).ti,ab,kw. (8553017)

799 (adenoma? or adenocarcinoma? or adeno-carcinoma? or blastoma? or carcinosarcoma? or carcino-sarcoma? or hepatoblastoma? or hepato-blastoma? or leukemia? or leukaemia? or lymphoma? or melanoma? or mesenchymoma? or mesothelioma? or sarcoma? or thymoma?).ti,ab,kw. (2073013)

800 exp Spinal Dysraphism/ (21584)

801 ((spina? or status) adj (bifida? or dysraphi*)).ti,ab,kw. (22190)

802 (((cleft or open) adj spine?) or rachischis* or schistorrhach*).ti,ab,kw. (440)

803 Child Development Disorders, Pervasive/ (32020)

804 (development* disorder? adj2 pervasive*).ti,ab,kw. (8324)

805 Mental Disorders/ (310497)

806 ((mental* or psychiatric*) adj1 (disorder? or ill* or disease?)).ti,ab,kw. (460872)

807 (behavio?r* adj1 disorder?).ti,ab,kw. (46308)

808 exp Mood Disorders/ (813276)

809 mood disorder?.ti,ab,kw. (64095)

810 Depression/ (531505)

811 depress*.ti,ab,kw. (1514880)

812 exp Schizophrenia/ (400024)

813 (dementia praecox or schizophreni*).ti,ab,kw. (446702)

814 exp Tic Disorders/ (21241)

815 (tic disorder? or facial twitch* or habit spasm* or nervous twitch*).ti,ab,kw. (5366)

816 tourette*.ti,ab,kw. (16977)

817 or/648-816 [CHRONIC CONDITIONS/DISABILITIES] (25248364)

818 647 and 817 [CHILDREN - CHRONIC CONDITIONS/DISABILITIES] (3763686)

819 639 or 818 [CHILDREN - CHRONIC CONDITIONS/DISABILITIES] (3766179)

820 exp Child Health Services/ (121260)

821 Child Day Care Centers/ (17683)

822 ((care or daycare or healthcare or program? or programme? or service?) adj3 (baby or babies or infant? or infanc* or neonat* or newborn* or preschool* or pre-school* or toddler* or child* or pre-adolescen* or preteen* or pre-teen* or school-age* or p?ediatric*)).ti,ab,kw. (382419)

823 ((intervention* or therap* or treatment* or rehab*) adj3 (baby or babies or infant? or infanc* or neonat* or newborn* or preschool* or pre-school* or toddler* or child* or pre-adolescen* or preteen* or pre-teen* or school-age* or p?ediatric*)).ti,ab,kw. (367851)

824 (early intervention* or head start*).ti,ab,kw. (75866)

825 School Health Services/ (34823)

826 School Mental Health Services/ (42)

827 School Nursing/ (10929)

828 (school? adj3 health service?).ti,ab,kw. (4541)

829 school nursing.ti,ab,kw. (2348)

830 or/820-829 [CHILD HEALTH SERVICES/THERAPIES] (890875)

831 Professional-Family Relations/ (97512)

832 ((care provider* or clinician* or doctor* or HCP or HCPs or physician* or professional* or provider* or therapist*) adj3 (family or families or parent*) adj5 relation*).ti,ab,kw. (3701)

833 Patient-Centered Care/ (205330)

834 ((family or families or parent*) adj3 (centre* or center$2 or support*)).ti,ab,kw. (121760)

835 ((patient or patients) adj3 (centre* or center$2 or support*)).ti,ab,kw. (297233)

836 collaborat*.ti,ab,kw. (461123)

837 (partnering or partnership?).ti,ab,kw. (116845)

838 ((care or healthcare or health care or service? or program? or programme? or relational) adj3 coordinat*).ti,ab,kw. (45835)

839 Patient Satisfaction/ (237760)

840 Patient Preference/ (28064)

841 Patient Participation/ (57022)

842 ((participat* or prefer* or satisf*) adj3 (family* or families or parent*)).ti,ab,kw. (63537)

843 ((participat* or prefer* or satisf*) adj3 (baby or babies or infant? or infanc* or neonat* or newborn* or preschool* or pre-school* or toddler* or child* or pre-adolescen* or preteen* or pre-teen* or school-age* or p?ediatric*)).ti,ab,kw. (71196)

844 ((participat* or prefer* or satisf*) adj3 patient*).ti,ab,kw. (363419)

845 Decision Making, Shared/ (7471)

846 (decision* adj3 (engag* or participat* or partner* or share? or sharing or active* involv*)).ti,ab,kw. (45466)

847 or/831-846 [FAMILY-CENTRED] (1865534)

848 830 and 847 [FAMILY-CENTRED CHILD HEALTH SERVICES] (105876)

849 819 and 848 [CHILDREN - CHRONIC CONDITIONS/DISABILITIES - FAMILY-CENTRED CHILD HEALTH SERVICES] (46744)

850 limit 849 to yr="2010-current" [Limit not valid in DARE; records were retained] (30891)

851 conference abstract.pt. (3860418)

852 journal conference abstract.pt. (158315)

853 850 not (851 or 852) [CONFERENCE ABSTRACTS REMOVED] (24183)

854 853 use coch,cctr,dare,clhta,cleed [COCHRANE DATABASES] (3265)

855 218 or 431 or 638 or 854 [ALL DATABASES] (24430)

856 limit 855 to yr="2019-current" [Limit not valid in DARE; records were retained] (4785)

857 remove duplicates from 856 (2985)

858 limit 855 to yr="2017-2018" [Limit not valid in DARE; records were retained] (5493)

859 remove duplicates from 858 (3407)

860 limit 855 to yr="2015-2016" [Limit not valid in DARE; records were retained] (4827)

861 remove duplicates from 860 (3021)

862 limit 855 to yr="2014-2015" [Limit not valid in DARE; records were retained] (4675)

863 remove duplicates from 862 (2873)

864 limit 855 to yr="2012-2013" [Limit not valid in DARE; records were retained] (3871)

865 remove duplicates from 864 (2390)

866 limit 855 to yr="2010-2011" [Limit not valid in DARE; records were retained] (3237)

867 remove duplicates from 866 (2010)

868 857 or 859 or 861 or 863 or 865 or 867 [TOTAL UNIQUE RECORDS] (15183)

869 868 use medall [MEDLINE UNIQUE RECORDS] (7497)

870 868 use emczd [EMBASE UNIQUE RECORDS] (2638)

871 868 use medall,emczd,coch,cctr,dare,cleed,clhta (12603)

872 868 not 871 [PSYCINFO UNIQUE RECORDS] (2580)

873 868 use cctr [CENTRAL UNIQUE RECORDS] (2385)

874 868 use coch [COCHRANE UNIQUE RECORDS] (72)

875 868 use dare [DARE UNIQUE RECORDS] (7)

876 868 use clhta [HTA UNIQUE RECORDS] (1)

877 868 use cleed [NHS EED UNIQUE RECORDS] (3)

***************************
