# Supplementary material for: Family‐centred care interventions for children with chronic conditions: A scoping review
Source: Health Expect. 2024 Feb 2;27(1):e13897. doi: 10.1111/hex.13897 (PMC10837485; doi:10.1111/hex.13897)
Supplement: Supplementary file 3 — Supporting information. [file HEX-27-e13897-s001.docx]

**Appendix 3. Extracted data items**

| **Bibliographical Information** |
| --- |
| RefID |
| Author |
| Title |
| Journal |
| Publication Year |
| Country (data collection) |
| **Study and Sample Characteristics** |
| Study design |
| Study objective |
| Sample size |
| Type of study participants |
| Age of child - eligible |
| Health care need of child - eligible |
| Age of child – sample |
| Health care need of child - sample |
| **Intervention Characteristics** |
| Name |
| Description |
| Objective |
| Rationale, theory |
| WHO: Planning/development |
| HOW: Planning/development |
| Author-developed or adapted |
| Materials – what, where |
| Procedures / activities |
| Type of health care need (targeted by intervention) |
| Intended recipients |
| Facilitator |
| Mode of delivery |
| Setting |
| Time period |
| Number of times intervention delivered |
| Number of sessions/contacts/interactions within intervention |
| Duration or intensity (schedule) |
| Tailoring |
| Modifications |
| **Family-centred care aspects (Picker Principles)** |
| For each domain, extract a) goals or activities and b) outcomes |
| **Domains** |
| Access to care |
| Communication and information provision |
| Family involvement |
| Care coordination |
| Physical comfort |
| Emotional support |
| Respect for child and family |
| Follow-up and continuity of care |
