# Supplementary material for: Family‐centred care interventions for children with chronic conditions: A scoping review
Source: Health Expect. 2024 Feb 2;27(1):e13897. doi: 10.1111/hex.13897 (PMC10837485; doi:10.1111/hex.13897)
Supplement: Supplementary file 4 — Supporting information. [file HEX-27-e13897-s007.docx]

**Appendix 4. Family-centred care domain definitions**

| **Family-centred care domain** | **Definition** |
| --- | --- |
| Communication and information provision | Provide information:   - On clinical status, progress and prognosis - On processes of care - To facilitate autonomy, self-care and health promotion |
| Family involvement | Provide accommodations for family and friends  Involve family and close friends in decision making  Support family members as caregivers  Recognize the needs of family and friends |
| Access to care | Access to the location of hospitals, clinics and physician offices  Availability of transportation  Ease of scheduling appointments  Availability of appointments when needed  Accessibility to specialists or specialty services when a referral is made  Clear instructions were provided on when and how to get referrals |
| Care coordination | Coordination of clinical care  Coordination of ancillary and support services  Coordination of front-line patient care |
| Respect for child and family | Involve patients in decision-making, recognizing they are individuals with their own unique values and preferences  Treat patients with dignity, respect and sensitivity to his/her cultural values and autonomy |
| Follow-up and continuity of care | Provide understandable, detailed information regarding medications, physical limitations, dietary needs, etc.  Coordinate and plan ongoing treatment and services after discharge  Provide information regarding access to clinical, social, physical and financial support on a continuing basis |
| Emotional support | Provide support for:   - Anxiety over physical status, treatment and prognosis - Anxiety over the impact of the illness on themselves and their family - Anxiety over the financial impact of illness |
| Physical comfort | Pain management  Assistance with activities and daily living needs  Hospital surroundings and environment |
