# Supplementary material for: Family‐centred care interventions for children with chronic conditions: A scoping review
Source: Health Expect. 2024 Feb 2;27(1):e13897. doi: 10.1111/hex.13897 (PMC10837485; doi:10.1111/hex.13897)
Supplement: Supplementary file 7 — Supporting information. [file HEX-27-e13897-s004.docx]

**Appendix 7. TIDieR checklist counts**

**Counts by TIDieR checklist item**

| **TIDieR checklist**  **item #** | **TIDieR checklist**  **item name** | **Reported** | **Underreported** | **Not reported** |
| --- | --- | --- | --- | --- |
| 1 | BRIEF NAME | 57 | 0 | 0 |
| 2 | WHY | 57 | 0 | 0 |
| 3 | WHAT | 18 | 27 | 12 |
| 4 | WHAT | 57 | 0 | 0 |
| 5 | WHO PROVIDED | 50 | 0 | 7 |
| 6 | HOW | 16 | 35 | 6 |
| 7 | WHERE | 51 | 0 | 6 |
| 8 | WHEN and HOW MUCH | 23 | 21 | 13 |
| 9 | TAILORING | 22 | 0 | 35 |
| 10 | MODIFICATIONS | 2 | 0 | 55 |

**Counts of reported TIDieR checklist item by intervention**

| **Study ID** | **Reported Items** | **Underreported Items** | **Not reported Items** |
| --- | --- | --- | --- |
| Alsem et al. 2019 | 4 | 2 | 4 |
| An et al. 2019 a,b | 9 | 0 | 1 |
| Barriteau et al. 2020 | 6 | 0 | 4 |
| Bosak et al. 2019 | 4 | 2 | 4 |
| Callahan et al. 2019 | 7 | 2 | 1 |
| Cama et al. 2020 | 5 | 1 | 4 |
| Camden 2019 | 5 | 3 | 2 |
| Caskey et al. 2019 | 6 | 2 | 2 |
| Chakravorty et al. 2019 | 5 | 2 | 3 |
| Cho et al. 2019 | 5 | 2 | 3 |
| Clark et al. 2019 | 7 | 2 | 1 |
| Dadds et al. 2019 | 6 | 1 | 3 |
| Dean et al. 2019 | 6 | 2 | 2 |
| Di Giuseppe et al. 2020 | 7 | 1 | 2 |
| Donnelly et al. 2020 | 5 | 2 | 3 |
| Eberhart et al. 2019 | 5 | 1 | 4 |
| El-Shanawany et al. 2019 | 6 | 2 | 2 |
| Feehan et al. 2020 | 6 | 2 | 2 |
| Feeley et al. 2020 | 4 | 1 | 5 |
| Fortini et al. 2020 | 6 | 1 | 3 |
| Geng et al. 2019 | 7 | 1 | 1 |
| Gilljam et al. 2020 | 6 | 2 | 2 |
| Green et al. 2019 | 5 | 2 | 3 |
| Hendricks-Ferguson and Haase 2019 | 8 | 1 | 1 |
| Hsieh et al. 2020 | 8 | 1 | 1 |
| Jacob et al. 2019 | 7 | 1 | 2 |
| Jerome et al. 2019 | 7 | 2 | 1 |
| Keetley et al. 2020 | 5 | 0 | 5 |
| King et al. 2019 | 5 | 0 | 5 |
| Kofoed and Thomsen 2019 | 7 | 1 | 2 |
| Kolko et al. 2020; Hsiung et al. 2019 | 7 | 2 | 1 |
| Kumar et al. 2019 | 5 | 3 | 2 |
| Lachal et al. 2019 | 6 | 1 | 3 |
| Lawson et al. 2020 | 9 | 1 | 0 |
| Lindstrom et al. 2020 | 9 | 0 | 1 |
| Mayan et al. 2020 | 7 | 0 | 3 |
| McRoberts et al. 2019 | 8 | 1 | 1 |
| Miller et al. 2019 | 7 | 1 | 2 |
| Ming et al. 2019 | 4 | 2 | 4 |
| Moeenuddin et al. 2019 | 5 | 3 | 2 |
| Mutambo et al. 2020 | 5 | 2 | 3 |
| Nagae et al. 2019 | 5 | 1 | 4 |
| Niemann et al. 2020 | 6 | 1 | 3 |
| Niemitz et al. 2019 | 7 | 1 | 2 |
| Nilses et al. 2019 | 8 | 1 | 1 |
| Nkoy et al. 2019 | 7 | 2 | 1 |
| Parikh et al. 2020 | 5 | 2 | 3 |
| Patel et al. 2019 | 5 | 2 | 3 |
| Sadof et al. 2019 | 7 | 2 | 1 |
| Santos Malavé et al. 2019 | 7 | 1 | 2 |
| Sleath et al. 2019 | 6 | 1 | 3 |
| Trace et al. 2020 | 5 | 2 | 3 |
| Uhm and Kim 2019 | 8 | 1 | 1 |
| Vusio et al. 2020 | 5 | 2 | 3 |
| Wihak et al. 2020 | 7 | 2 | 1 |
| Yamada et al. 2020 | 8 | 0 | 2 |
| Young et al. 2019 | 8 | 1 | 1 |
