# Supplementary material for: Family‐centred care interventions for children with chronic conditions: A scoping review
Source: Health Expect. 2024 Feb 2;27(1):e13897. doi: 10.1111/hex.13897 (PMC10837485; doi:10.1111/hex.13897)
Supplement: Supplementary file 8 — Supporting information. [file HEX-27-e13897-s009.docx]

**Appendix 8. Map of interventions addressing family-centred care domains**

| **Author, yr, citation** | **Communication & information provision** | **Family involvement** | **Access to care** | **Care coordination** | **Respect for child & family** | **Follow-up & continuity of care** | **Emotional support** | **Physical comfort** |
| --- | --- | --- | --- | --- | --- | --- | --- | --- |
| Alsem et al. 2019 | ✓ | ✓ | No | No | No | No | No | No |
| An et al. 2019; An et al. 2018 | ✓ | ✓ | No | No | ✓ | No | No | No |
| Barriteau et al. 2020 | ✓ | No | ✓ | ✓ | No | No | No | No |
| Beck et al. 2019 | ✓ | ✓ | No | No | ✓ | No | No | No |
| Bosak et al. 2019 | No | ✓ | No | No | ✓ | No | No | No |
| Callahan et al. 2019 | No | ✓ | No | No | No | No | ✓ | No |
| Cama et al. 2020 | No | No | ✓ | ✓ | No | No | No | No |
| Camden 2019 | ✓ | No | ✓ | No | No | No | No | No |
| Caskey et al. 2019 | ✓ | ✓ | ✓ | ✓ | No | ✓ | No | No |
| Chakravorty et al. 2019 | ✓ | ✓ | ✓ | ✓ | ✓ | No | No | No |
| Cho et al. 2019 | ✓ | No | ✓ | ✓ | No | ✓ | No | No |
| Clark et al. 2019 | ✓ | ✓ | ✓ | ✓ | ✓ | ✓ | ✓ | ✓ |
| Dadds et al 2019 | ✓ | ✓ | ✓ | No | No | No | ✓ | No |
| Dean et al. 2019 | No | No | ✓ | No | No | No | No | No |
| Di Giuseppe et al. 2020 | ✓ | No | No | No | No | No | No | No |
| Donnelly et al. 2020 | ✓ | ✓ | ✓ | ✓ | ✓ | ✓ | ✓ | No |
| Eberhart et al 2019 | ✓ | ✓ | ✓ | No | ✓ | No | No | No |
| El-Shanawany et al. 2019 | No | No | ✓ | ✓ | No | ✓ | No | No |
| Feehan et al. 2020 | ✓ | ✓ | ✓ | ✓ | ✓ | ✓ | ✓ | No |
| Feeley et al. 2020 | ✓ | ✓ | No | No | No | No | ✓ | ✓ |
| Fortini et al. 2020 | No | No | ✓ | No | No | No | No | No |
| Geng et al. 2019 | ✓ | No | ✓ | No | No | ✓ | ✓ | No |
| Gilljam et al. 2020 | ✓ | No | No | No | ✓ | No | ✓ | ✓ |
| Green et al. 2019 | No | No | ✓ | ✓ | No | No | No | No |
| Hendricks-Ferguson & Haase 2019 | ✓ | ✓ | No | No | ✓ | No | No | No |
| Hsieh et al. 2020 | ✓ | ✓ | No | No | No | No | No | No |
| Jacob et al. 2019 | No | No | No | ✓ | No | No | No | No |
| Jerome et al. 2019 | No | ✓ | No | No | No | No | No | No |
| Keetley et al. 2020 | No | No | No | ✓ | No | ✓ | No | No |
| King et al. 2019 | No | ✓ | No | No | ✓ | No | No | No |
| Kofoed and Thomsen 2019 | No | No | ✓ | ✓ | No | ✓ | No | No |
| Kolko et al. 2020; Hsiung et al. 2019 | ✓ | ✓ | ✓ | ✓ | No | ✓ | No | No |
| Kumar et al. 2019 | ✓ | ✓ | No | No | No | No | ✓ | ✓ |
| Lachal et al. 2019 | ✓ | ✓ | No | ✓ | ✓ | No | No | No |
| Lawson et al. 2020 | ✓ | ✓ | No | No | ✓ | No | No | No |
| Lindstrom et al 2020 | ✓ | ✓ | No | No | No | ✓ | No | No |
| Lyon et al. 2019 | ✓ | ✓ | No | No | ✓ | ✓ | ✓ | No |
| Mayan et al. 2020 | No | ✓ | No | No | ✓ | No | ✓ | No |
| McRoberts et al. 2019 | ✓ | No | No | ✓ | ✓ | ✓ | No | No |
| Miller et al. 2019 | No | No | No | No | No | No | ✓ | ✓ |
| Ming et al. 2019 | ✓ | ✓ | No | ✓ | No | No | No | No |
| Moeenuddin et al. 2019 | No | No | ✓ | ✓ | No | ✓ | No | No |
| Mutambo et al. 2020 | ✓ | No | No | No | ✓ | No | ✓ | ✓ |
| Nagae et al. 2019 | ✓ | ✓ | No | No | ✓ | No | No | No |
| NICE 2019a | ✓ | ✓ | ✓ | ✓ | ✓ | ✓ | ✓ | No |
| NICE 2019b | ✓ | ✓ | No | ✓ | ✓ | ✓ | ✓ | No |
| NICE 2019c | ✓ | ✓ | ✓ | ✓ | ✓ | ✓ | ✓ | ✓ |
| Niemann et al. 2020 | ✓ | ✓ | ✓ | ✓ | No | ✓ | No | No |
| Niemitz et al. 2019 | ✓ | ✓ | No | No | ✓ | No | No | No |
| Nilses et al. 2019 | ✓ | ✓ | ✓ | ✓ | No | ✓ | ✓ | No |
| Nkoy et al. 2019 | ✓ | ✓ | No | No | No | No | No | No |
| Parikh et al. 2020 | ✓ | No | ✓ | ✓ | No | ✓ | No | No |
| Patel et al. 2019 | ✓ | No | No | No | ✓ | No | No | No |
| Sadof et al. 2019 | No | ✓ | ✓ | ✓ | ✓ | ✓ | ✓ | No |
| Santos Malavé et al. 2019 | ✓ | No | ✓ | ✓ | ✓ | ✓ | No | No |
| Sleath et al. 2019 | ✓ | ✓ | No | No | ✓ | No | No | No |
| Trace et al. 2020 | ✓ | No | ✓ | No | No | No | No | No |
| Uhm and Kim 2019 | ✓ | ✓ | No | No | ✓ | No | No | No |
| Vusio et al. 2020 | ✓ | ✓ | ✓ | ✓ | No | ✓ | No | No |
| Wihak et al 2020 | ✓ | ✓ | ✓ | No | ✓ | No | ✓ | No |
| Yamada et al. 2020 | ✓ | No | No | ✓ | No | ✓ | No | No |
| Young et al. 2019 | ✓ | No | ✓ | No | No | ✓ | No | No |

✓: The intervention addressed the patient experience domain through its aims and objectives (orange) and/or activities (blue).
