# Supplementary material for: Family‐centred care interventions for children with chronic conditions: A scoping review
Source: Health Expect. 2024 Feb 2;27(1):e13897. doi: 10.1111/hex.13897 (PMC10837485; doi:10.1111/hex.13897)
Supplement: Supplementary file 9 — Supporting information. [file HEX-27-e13897-s003.docx]

**Appendix 9. List of potentially relevant articles excluded due to publication in languages other than English**

1. Kaulfersch W, Pichler E, Flaschberger S, et al. 20 years of outpatient homeopathic treatment in the Klagenfurt General Hospital. *Monatsschrift Kinderheilkunde.* 2019;167(9):788–95. doi: 10.1007/s00112-019-0739-8
2. Arnaud M, Baduel S, Rogé B, et al. Supporting families with emerging telehealth. *Enfance.* 2019; 2019(1):99–118.
3. Oelkers-Ax R, Heineken K. Family psychiatry - The attachment-focused, systemic-oriented, integrative concept of the Family Therapy Centre (FaTC), an acute multi-family day clinic. *Prax Kinderpsychol Kinderpsychiatr.* 2019;68(5):419–37. doi: 10.13109/prkk.2019.68.5.419
4. Bebchuk MA, Khodyreva LA, Basova AY, et al. Art therapy in treatment, rehabilitation, micro- and macrosocial adaptation of children with autism spectrum disorders by means of a special rehabilitation and adaptation program “Art therapy (drama therapy): ‘because you are needed…’ for children with general disorders of psychological development and other mental disorders.” *Probl Sotsialnoi Gig Istor Med*. 2019;27(Special Issue):536–42. doi: 10.32687/0869-866X-2019-27-si1-536-542
5. Ortiz J, Schepper F, Buttstädt M, et al. Family oriented art therapy for children and adolescents with cancer and their parents in the acute care setting. *Onkologe (Berl).* 2018;25(6):529–539. doi:
6. Dionne C, Paquet A, Boutet M. Approach to improve behavioral intensive intervention practices by a research-practice partnership: Reconciling best practices and transferability in real situation. *ANAE - Approche Neuropsychol. Apprentiss. chez Enfant.* 2019; 31(162):631-39.
7. Jabalera M, Pons M, Gómez E, et al. Towards excellence in hospital management. A description of strategical management model. *J Healthc Qual Res.* 2019;34(3):148–153. doi: 10.1016/j.jhqr.2019.02.005
8. Görtz-Dorten A, Hanisch C, Hautmann C, et al. The prevention of externalizing disturbances. Z *Kinder Jugendpsychiatr Psychother*. 2020;48(6):459–68. doi: 10.1024/1422-4917/a000650
9. Gonzalez Betlza M, Bruno I, Yemini L, et al. Impact of a program of health education for the asthmatic child and their families. *Arch Argent Pediatr.* 2020;118(2):145–149. doi: 10.5546/aap.2020.145
